# Establishment of the mechanism of purification and levigation of green chemistry-assisted biocomposites of red ochre (Gairika): synthesis, characterization, and antibacterial, prebiotic, antioxidant, and antacid activities of the traditional Ayurvedic medicine Laghu Sutashekhara Rasa

**DOI:** 10.3389/fchem.2023.1271157

**Published:** 2023-11-23

**Authors:** Vaibhav Charde, Vijay Kumar, Ganesh Dane, Yashika Gandhi, Hemant Soni, Chandrashekar Jagtap, Sujeet K. Mishra, Santosh K. Shakya, Arjun Singh, Ravindra Singh, Bhagwan S. Sharma, Shruti Khanduri, Narayanam Srikanth, Rabinarayan Acharya, Thomas J. Webster

**Affiliations:** ^1^ Central Ayurveda Research Institute, Jhansi, Uttar Pradesh, India; ^2^ Central Council for Research in Ayurvedic Sciences, New Delhi, India; ^3^ School of Health Sciences and Biomedical Engineering, Hebei University of Technology, Tianjin, China; ^4^ School of Engineering, Saveetha University, Chennai, India; ^5^ Program in Materials Science, UFPI, Teresina, Brazil

**Keywords:** Gairika, red ochre, Laghu Sutashekhara Rasa, hematite, goethite, clay

## Abstract

Gairika (red ochre) has a long history of influencing human civilization. Gairika is a rich source of nutrients used for reproductive and brain health. Gairika is mentioned as an antacid drug in Indian Ayurvedic medicine under Laghu Sutashekhara Rasa (LSR). However, a detailed study on LSR has not been reported to date. In the present study, LSR was prepared, and a pharmaceutical SOP (standardization procedure) was reported to obtain batch-to-batch reproducibility. LSR was characterized using FTIR, XRD, SEM-EDX, and TGA analyses. LSR was tested *in vitro* for its antacid activity. Advanced instrumentation revealed that LSR formation produced symmetrical particles (5–8 µm) with kaolin, kaolinite, quartz, goethite, and hematite, along with the phytoconstituents of Goghrita (clarified cow’s butter), Shunthi, and Nagawalli, as confirmed by GC-MS/MS analysis. The FTIR study revealed the formation of a chelating complex of goethite and hematite along with their phytoconstituents. XRD analysis confirmed the presence of kaolin, kaolinite, quartz, goethite, and hematite. Using *in vitro* antacid experiments, LSR and Shunthi demonstrated significant antacid activity as compared to antacid drugs and standards in the market, such as CaCO_3_. The DPPH assay revealed IC_50_ values of 12.16 ± 1.23 mg/mL, which is 0.0029 of Trolox-equivalent antioxidant activity. The inhibition (18 ± 4 mm) against pathogens (*S*. *aureus*, *E. coli*, *P. aeruginosa*, and *B*. *subtilis*) and the prominent growth of gut microbiota-supported strains (*S. boulardii*, *L*. *paracasei*, and *L. plantarum*) observed on LSR formulation were indicative of LSR application as a prebiotic. Here, the mechanism of purification and levigation mentioned in the classical literature of LSR was established. Overall, purification of Gairika with cow ghee and levigation with Nagawalli may enhance the solubility, bioavailability, and shelf-life of LSR through hydration and co-crystallization mechanisms. This is the first comprehensive report on the pharmaceutical validation of LSR and its characterization. The results of the present study could contribute to the development and reliable reproduction of LSR and the utility of environmental red ochre as a medicine in combination with Shunthi (*Zingiber officinale* Roxb.), as prescribed under Indian Ayurvedic medicine.

## Graphical Abstract

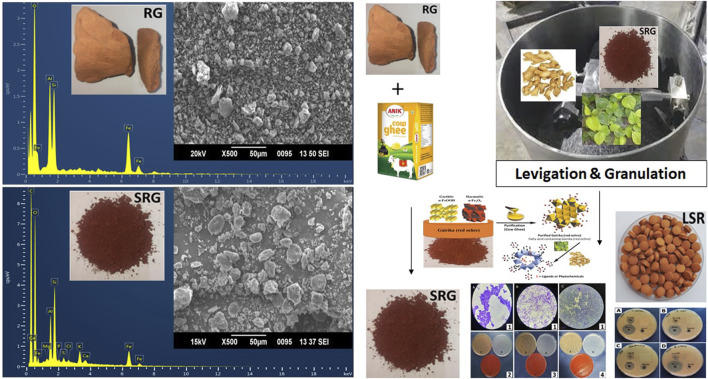

## 1 Introduction

Clay and iron oxides, including Gairika (red ochre), are the best-ever gift from the environment, as these are used in environment management and medicine preparation (Carretero et al., 2013; Hradil et al., 2017). Iron oxides (red ochre) are known as a natural pigment with minimal side effects. The macro- and nanoformulations of Gairika (red ochre) are used to clean the environment and in medicine, the art and craft world, agriculture, ship maintenance, and archeology (Jones et al., 2018; Teklay et al., 2023). Due to its UV-protection ability, red ochre has been used in skin care applications and is approved by the US FDA (Teklay et al., 2023). Overall, red ochre is the best-ever gift from the environment to humans, having cultural and medicinal applications (Teklay et al., 2023). Even minor modifications in the red ochre signature can alter its bioactivity and commercial applications, so the bioactivity of red ochre is considered more important than its red color (Jones et al., 2018).

In the natural habitat, Gairika is available in clay form. These types of clays are known for their medicinal properties, being strongly absorbant, and having been employed as antacids and antidiarrheal agents since antiquity (Hradil et al., 2017). Clays and clay soils bearing kaolinite, smectite, palygorskite, or iron (hydr)oxides are used the most to treat acidity and heartburn (Carretero et al., 2013). The antacid action of different types of clays, like smectites, palygorskite, sepiolite, kaolinite, and talc, is well known, but unexplored for Gairika, which was the aim of the current study. Red ochre has a direct link with human evolution. The medicinal applications of red ochre with seafood and herbs could be a source of nutrients for reproductive and brain health (Duarte, 2014).

The three major traditional medicinal practices globally are Ayurveda, traditional Chinese medicine, and Arabic medicine (Li et al., 2022). Iron-based medicines have great importance in the Indian medicine system (Krishnamachary et al., 2012; Farooq et al., 2019). Purified (shodhita) Gairika, iron oxide, and iron dust are used in Ayurveda, Tibetan medicine, and Buddhist medicinal preparations (Li et al., 2022). Iron-based Ayurvedic nanoparticles or Bhasma are used to treat anemia and anemia-related diseases (Pandit et al., 1999; Farooq et al., 2019). The shodhana (purification) of Gairika, iron oxide, and iron dust is a key factor for their potency (Krishnamachary et al., 2012; Chandra and Prakash, 2020).

As per the Indian Ayurvedic medicinal system, red ochre is used for the treatment of various diseases, including *Pittaja Shirashoola* (headache due to Pitta dosha), *Ardhavabhedaka* (migraine), *Suryavarta* (sinusitis), *Pittaja Unmada* (insanity due to Pitta dosha), *Daha* (burning sensation), *Urdhva Raktapitta* (bleeding from orifices in the upper part of the body), and *Mukhapaka* (stomatitis). There is a large scope for exploring the medicinal applications of red ochre. In a clinical study, red ochre was found to be potent in controlling excessive menstrual bleeding (Kotagasti, 2015). In the present study, *Daha* (a burning sensation or acidity) was undertaken and explored.

In the present study, the Ayurvedic herbo-mineral formulation Laghu Sutashekhara Rasa (LSR) was explored for its antacid activity. LSR is the combination of two parts of Shuddha Gairika (Fe_2_O_3_) and one part of Shunthi (*Zingiber officinale* Roxb.), with the levigation of Nagawalli Swarasa (fresh juice of *Piper betel Linn*.) (Shweta et al., 2020). This is an important formulation in Ayurveda therapeutics, especially when used as an antacid, but its preparation and antioxidant and antacid activity have not been reported until now.

## 2 Materials and methods

### 2.1 Collection of plant materials and authentication

The raw drugs, namely, Gairika (red ochre) and Goghrita (clarified cow’s butter), were procured from local vendors of Jhansi and analyzed for quality control parameters before their use. The raw drugs, namely, Shunthi (*Zingiber officinale* Roxb.) and Nagawalli Swarasa (*Piper betel Linn*.), were collected from the herbal garden of the Central Ayurveda Research Institute, Jhansi, and authenticated by a botanist (identification numbers JHS/ZO/123 & JHS/PB/129). The plant names were cross-checked with “World Flora Online” (www.worldfloraonline.org).

### 2.2 Chemicals and reagents

The standards, namely, 6-shagaol, pepsin, gallic acid, DPPH, Trolox, and CaCO_3_, of technical grade purity >95%, were procured from Sigma-Aldrich (Mumbai, India). The antacid drug (brand: Gelusil) was procured from a local market in Jhansi, India. All the reagents used in the present study were of AR grade, and the solvents were of HPLC grade. Goghrita (clarified cow’s butter from Anik brand) was procured from a local market in Jhansi, UP, India, and utilized in the formulations after complete analysis. Elemental standards (Al, As, Ca, Cd, Cu, Fe, Hg, K, Mg, Mn, Na, Pb, and Zn), nitric acid, sodium chloride, pepsin, hydrochloric acid, and calcium carbonate were procured from Merck. Analytical-grade safranin, fast green, phloroglucinol, Sudan red III, Dragendorff’s solution, ferric chloride, sulfuric acid, hydrochloric acid, sodium hydroxide, acetic acid, and iodine solution were procured from Loba Chemie.

Test strains, including Gram-negative pathogens (*Escherichia coli* (MTCC no. 1885), *Pseudomonas aeruginosa* (MTCC no. 424), and *Salmonella typhi* (MTCC no. 733)), a Gram-positive pathogen (*Staphylococcus aureus* (MTCC no. 1430)), and a probiotic bacterial strain (*Lactobacillus plantarum* (MTCC no. 1407)), were procured from MTCC Chandigarh, India. Culture media, including nutrient broth (NB), nutrient agar (NA), and Mueller–Hinton agar (MHA), were purchased from HiMedia Laboratories Private Limited, Thane, Maharashtra, India. Petri dishes (90 mm), the cork borer (6 mm), cotton swabs, and McFarland standards were also procured from HiMedia. The probiotic bacterial strain *Lactobacillus paracasei* (MCC no. 4490) was purchased from the Microbial Culture Collection (MCC), Pune, and *Saccharomyces boulardii* was isolated from commercially available probiotics purchased from a local market.

### 2.3 Purification (shodhana) of Gairika (red ochre), preparation of LSR, detailed QC parameters, essential metal ion analysis, and detailed pharmaceutical SOP preparation

The validation and preparation of a pharmaceutical standard operating procedure (SOP) and detailed quality control studies were completed. In brief, for the shodhana of red Gairika (RG), the lump-free, non-purified or raw RG was taken and grounded using a mortar and pestle and sifted through a mesh #200 using an SS Vibro Sifter. The stainless-steel vessel (kadhai) was heated up to 100°C and cow’s ghee was added to liquify it. The powdered RG was then slowly added. The ratio of Goghrita (clarified cow’s butter) to powdered red ochre was kept at 1:4 (w/w) for each batch (three batches were prepared, named as LSR-I, -II, and -III). The roasting process was continued until the powdered red ochre became free flowing and the ghee started fuming mildly. The temperature was maintained between 140°C–170°C and recorded every 5 min. The time required for the completion of the process and mass gains were recorded.

Finally, the material (shodhita Gairika, i.e., SRG) was self-cooled in a closed stainless-steel vessel. The Shunthi (*Zingiber officinale* Roxb.) powder with a sieve size of 80 was added to SRG at a 1:2 ratio and mixed well. The aforementioned mixture of Shunthi and SRG was levigated with Nagawalli Swarasa (*Piper betel Linn*. leaf extract) for 1 hour. After the levigation process, Babul Gond solution and lactose were added (up to the permissible quantities), and granules were prepared by passing the mixture through a granulator with sieve no. 10. Granule tablets were prepared using a Single Rotary Tablet Machine with 16 stations. The “In-Process Quality Control” (IPQC) parameters (like set size, weight, hardness, and friability of the tablet) were checked. The tablets were collected, weighed, and kept in a pharmaceutical-grade air-tight plastic container for further applications.

All the raw drugs, intermediates, and the final drug LSR were tested for their quality control parameters. The organoleptic, microscopic, physicochemical, heavy metal ion, aflatoxin, pesticide residue, microbial load, and pharmaceutical parameters were determined using advanced instrumentation (Kumar et al., 2022a). The identification of the major markers of raw drugs and the polyherbal formulation (LSR) was carried out using HPTLC analysis. LSR was characterized using FTIR, XRD, SEM-EDX, and TGA analyses as per the procedures mentioned in our recent article (Kumar et al., 2019d). The procedures used for HPTLC analysis were carried out as per our recent report and the detailed procedure mentioned in the Supplementary Material (Kumar et al., 2022b). Heavy-metal ions and essential metal ions of raw drugs and BM were analyzed by using ICP-OES as per our recent work (Kumar et al., 2019c).

### 2.4 Gas chromatography–mass spectrometry analysis of LSR

Gas chromatography–mass spectrometry (GC–MS/MS) analysis of the LSR tablet was carried out by employing an Agilent 8890 GC System together with a 7000D GC/TQ, using a combination of two Agilent HP-5ms UI columns (5% (phenyl)-methylpolysiloxane; non-polar; 15 m × 250 μm x 0.25 μm). The separation of the phytoconstituents present in the methanolic extract was carried out by employing helium as a carrier gas at constant flow rates of 1.0 mL/min and 1.1 mL/min in columns I and II, respectively. The methanolic extract solution (10 mg/mL) was injected with an injection volume of 1 μL and a split ratio of 3:1 into the column using an autosampler. During the chromatographic run, the initial oven temperature was 60°C, which was increased to 280°C at a rate of 5°C/min (at a hold time of 5 min). The conditions of the mass detector were as follows: a source temperature of 230°C, mass detector (MSD) transfer line temperature of 280°C, ionization mode electron impact (EI) of 70 eV, scan time of 300 ms (0.3 s), positive and MS2 scan modes with a solvent delay of 3 min, and a mass range of 50–650. The pressures of the collision gas (N_2_) and quenched gas (He) were set as 1.5 mL/min and 2.25 mL/min, respectively. The mass spectra of the major peaks obtained using the GC–MS chromatogram were compared with the database present in the GC–MS NIST library.

### 2.5 *In vitro* medicinal properties

The *in vitro* medicinal activities like antimicrobial, prebiotic, antioxidant, and antacid activities were examined using the modified reported methods. For antibacterial activities, the 100 mg/mL solution of LSR was used and methanol was used as a negative control along with the positive control kanamycin (1 mg/mL conc.), as prescribed by Balouiri et al., 2016. For prebiotic studies, a fine powder of LSR was used (Ribeiro et al., 2021). The detailed protocols of antibacterial and prebiotic studies are mentioned in the Supplementary Material. The antioxidant activity of LSR was evaluated using DPPH with Trolox as the positive control (i.e., Trolox-equivalent antioxidant activity (TEAC)) (Kumar et al., 2019d). The antacid activity of LSR was analyzed using Fordtran’s titration model (Fordtran et al., 1973; Christensen et al., 2015). In brief, the ground LSR samples (666 or 1,332 mg, 666 or 1,332 mg + 400 mg CaCO_3_, and 400 mg CaCO_3_) were placed in a 500-mL beaker containing tap water (90 mL), having a temperature of 37°C, and were stirred at 30 rpm using a magnetic stirrer (to mimic stomach movement). The sample in the beaker was titrated with artificial gastric acid (pepsin >4000 U/L, Sigma-Aldrich, in 34 mM NaCl and pH 1.20 adjusted using HCl) to an end point pH of 3 (Fordtran et al., 1973). The acid secretion rate was adjusted to 3 mL/min. All tests were performed in triplicates.

### 2.6 Statistical analysis

All the experiments were performed in triplicate and expressed as the mean. Student’s *t*-test and one-way ANOVA tests were applied to check the significant difference between the control and the sample or the treated and the untreated group at *p* <0.05. Origin 2019 and Excel 2021 software were used for statistical analyses and to draw the plots.

## 3 Results and discussion

### 3.1 Purification (shodhana) of Gairika (red ochre), preparation of LSR, detailed QC parameters, essential metal ion analysis, and detailed pharmaceutical SOP preparation

The important step for the preparation of any formulation is its batch-to-batch reproducibility. This reproducibility depends on observations of minute-to-minute changes that occur during preparation. During the preparation of LSR and shodhana of RG, the main observations were as follows:

• The average time required for pulverizing and sifting RG was 3 h for each batch
• No sound was produced when grounded in a mortar with a pestle
• SRG (shodhita Gairika or purified one) was soft to the touch when rubbed between the fingers and free flowing
• RG was converted into SRG (shodhita Gairika or purified one) and was sweet and astringent, like the clay odor characteristics of ghee
• Care should be taken to avoid sticking of GR in a stainless-steel vessel (kadhai) while heating with Goghrita (clarified cow’s butter)

**FIGURE 1:**
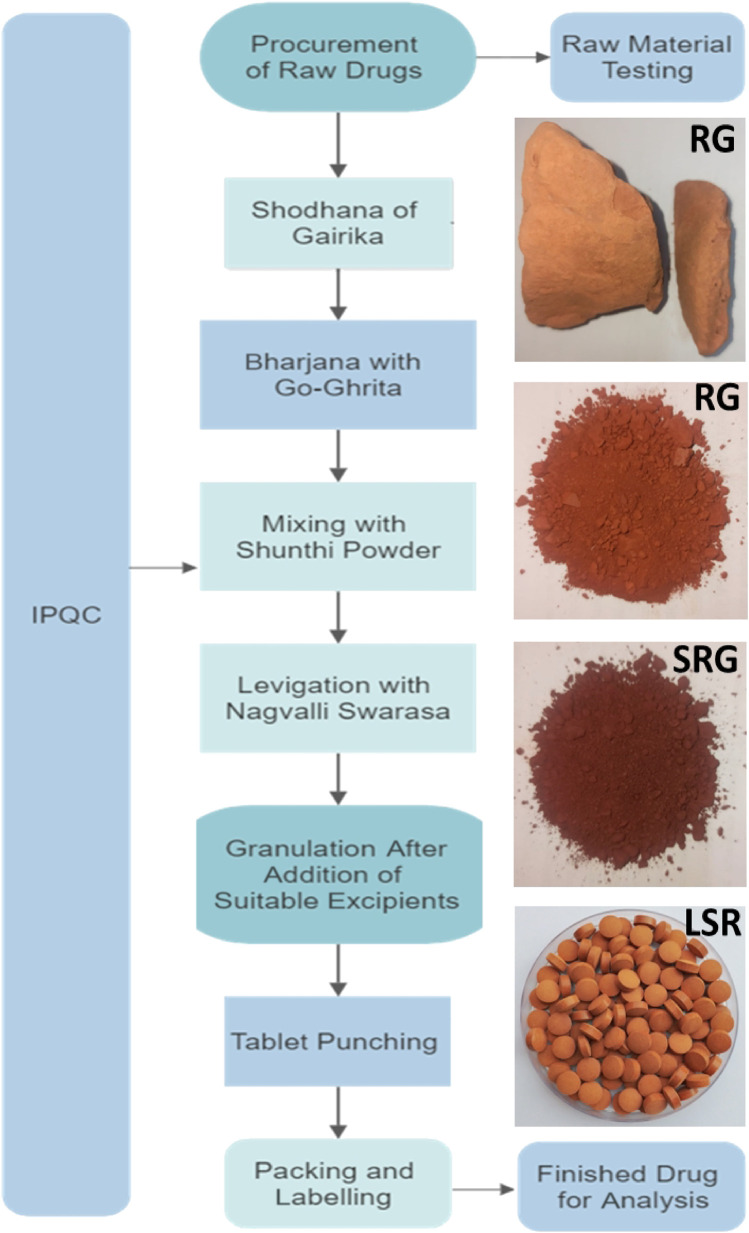
Brief SOP for the preparation of LSR.

The quality control data from three batches of LSR are mentioned under Table 1 and Figure 1. The quality control data of all raw drugs and IPQC are mentioned in the Supplementary Material. The data under Table 1 exhibited batch-to-batch reproducibility. The average weight of LSR tablets was 400 ± 5 mg. The percentage content of ash was on the higher side due to the presence of the metallic ore RG. The alcohol extract values were more than 10% w/w, indicating the presence of Shunthi and the proper levigated process with Nagawalli. Similarly, the water extractive values were more than 40% w/w, due to the slight solubility of the ore and the presence of Shunthi and Nagawalli. There was a significant amount of metal ions, namely, Na (2,100 ± 150 ppm), K (7,900 ± 170 ppm), Ca (8,300 ± 250 ppm), Mg (2,700 ± 100 ppm), Al (23,000 ± 700 ppm), and Fe (31,500 ± 500 ppm), which were all found in all three batches of LSR. The presence of these ions confirmed the availability of kaolin, kaolinite, quartz, goethite, and hematite in the LSR formulation, which was cross-verified with XRD and SEM-EDX analyses. The amount (percentage) of Na, K, Ca, Al, and Mg increased in LSR as compared to that in RG and SRG due to the elemental compositions of Shunthi and Nagvel. Moreover, the amount (percentage) of Fe (68% for SRG to 80% for RG) decreased in LSR as compared to that in RG and SRG due to the addition of Goghrita (clarified cow’s butter), Shunthi (one part), and levigation of Nagvel. The presence of Ca, Al, and Ca along with the other elements makes LSR more potent toward antacid activity (Carretero et al., 2013). HPTLC was the technique which was used to analyze herbal and herbo-mineral drugs. In the HPTLC analysis of LSR, with the developing solvent (toluene:ethyl acetate (3:1)), the LSR showed 6-gingerol (Rf ≈ 0.295). The percentage content of 6-gingerol (6G) in LSR was 0.13% ± 0.05% (Supplementary Figure S1).

**TABLE 1:** Physicochemical analysis of LSR tablets.

S. no.	Test(s)	LSR-I	LSR-II	LSR-III
1	Size	Diameter: 9.61 ± 0.14 mm; thickness: 4.76 ± 0.08 mm		
2	Foreign matter (% w/w)	**Nil**	**Nil**	**Nil**
3	Loss on drying (% w/w)	3.87 ± 0.47	3.55 ± 0.52	3.69 ± 0.42
4	pH (10% aqueous solution)	5.33 ± 0.65	5.36 ± 0.59	5.33 ± 0.68
5	Water extractive value (% w/w)	44.52 ± 2.21	43.08 ± 2.32	45.07 ± 2.37
6	Alcohol extractive value (% w/w)	12.34 ± 1.07	12.07 ± 1.12	11.88 ± 1.21
7	Total ash value (% w/w)	21.98 ± 1.55	22.28 ± 1.47	22.42 ± 1.58
8	Acid insoluble ash value (% w/w)	17.70 ± 1.14	17.62 ± 1.21	18.13 ± 1.17
9	Average weight (g)	0.402 ± 0.04	0.403 ± 0.06	0.405 ± 0.07
10	Hardness (kg/cm^2^)	4.34 ± 0.24	4.40 ± 0.27	4.75 ± 0.22
11	Disintegration time (min.)	17.16 ± 1.21	17.21 ± 1.25	17.43 ± 1.27
12	Friability (% w/w)	0.45 ± 0.05	0.46 ± 0.04	0.46 ± 0.04
13	Na (ppm)	2,214.87 ± 5.14	2,092.85 ± 5.21	2,124.55 ± 5.27
14	K (ppm)	7,925.10 ± 11.31	8,078.63 ± 11.87	7,844.19 ± 12.54
15	Ca (ppm)	8,312.22 ± 14.55	8,595.75 ± 14.61	8,213.48 ± 14.87
16	Mg (ppm)	2,878.78 ± 5.91	2,789.08 ± 5.88	2,621.91 ± 5.89
17	Al (ppm)	23,217.84 ± 21.87	23,006.11 ± 22.54	23,603.59 ± 22.65
18	Fe (ppm)	31,583.18 ± 24.87	31,432.04 ± 26.87	31,645.45 ± 25.71
19	Test for heavy metals (ppm)	Complies as per the API		
	(Pb, Cd, Hg, and As)			
20	Aflatoxins	Complies as per the API		
21	Specific pathogens	Complies as per the API		
22	Pesticide residues	Complies as per the API		

### 3.2 Elemental (CHNS) analysis

The elemental analysis of RG, SRG, and LSR was performed with C (carbon), H (hydrogen), N (nitrogen), and S (sulfur), where no S content was observed in all three samples. In the case of RG, only N (0.08%) was observed; the absence of C and H indicated the purity of RG and the absence of organic and environmental impurity. For SRG, the observed C, H, and N percentage was 15.24, 2.11, and 0.12%, respectively. For LSR, the observed CHN percentage was 26.93, 4.99, and 0.49%, respectively. Overall, CHNS analysis revealed that the percentage content of C, H, and N increased from the shodhana to the LSR preparation stage. The observed percentage of C, H, N, and S for the Shunthi (*Zingiber officinale*) rhizome was 31.76, 5.68, 1.23, and 0.83%, respectively. The observed percentage of C, H, N, and S for the Nagawalli (*Piper betel* Linn.) leaf was 31.25, 4.97, 2.80, and 0.15%, respectively.

### 3.3 FTIR analysis

**FIGURE 2:**
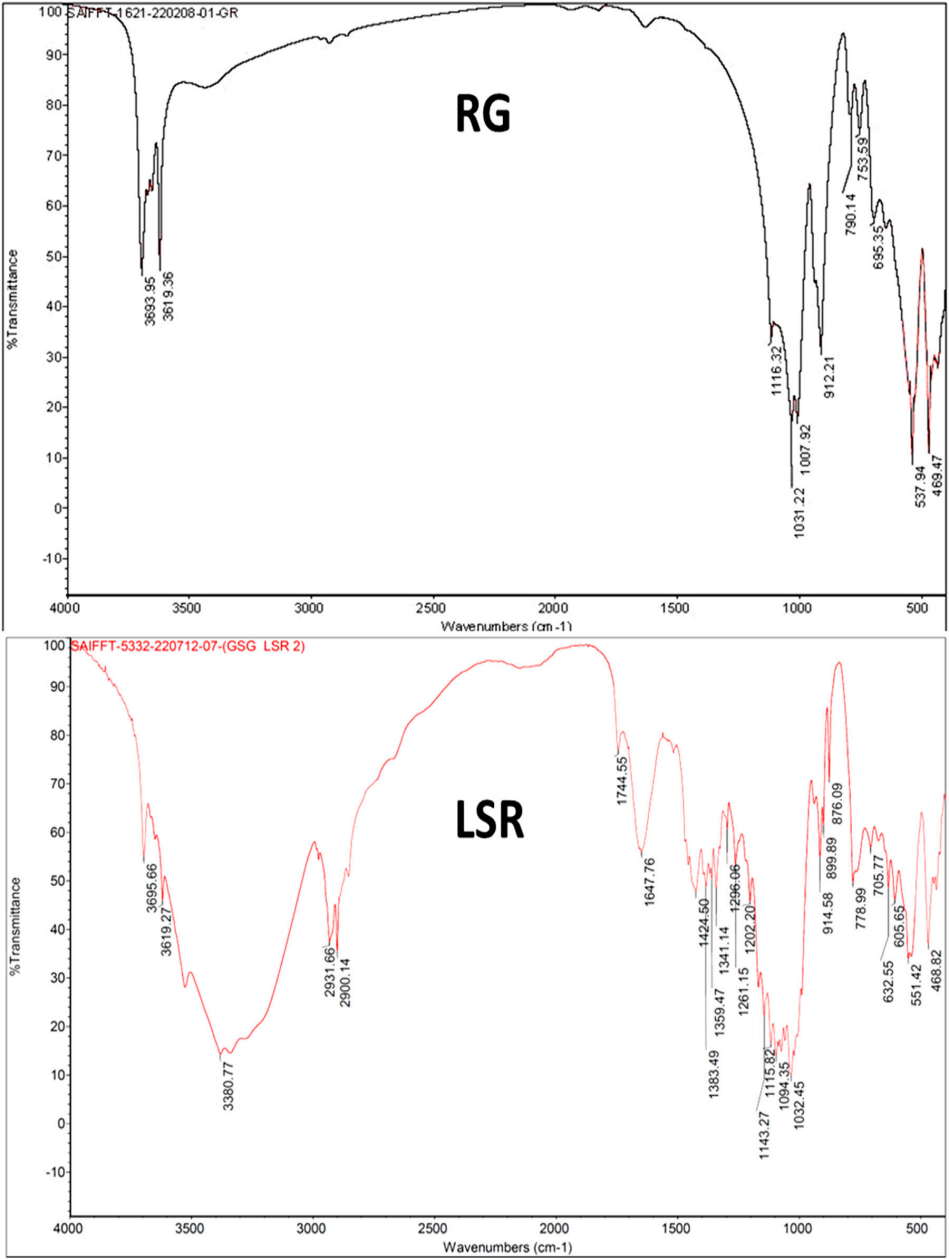
FTIR analysis of raw RG and LSR.

The FTIR spectrum of GR showed peaks for the O–H stretching bands of kaolin (Al_2_Si_2_O_5_(OH)_4_) at 3,693 and 3,619 cm^−1^. The asymmetric Si–O–Si stretching band, Si–O stretching band, and Si–O–Al vibration of kaolin were observed at 1,007, 912, and 753 and 694 cm^−1^, respectively. The Si–O antisymmetric stretching vibration of kaolinite was observed at 1,031 cm^−1^. The Si–O symmetrical stretching vibration of quartz (SiO_2_) was observed at 790 cm^−1^. The Fe–O vibrations of Fe_2_O_3_ were observed at 537 and 469 cm^−1^. The spectrum of GR almost resembled that of a recent study of Gairika performed by Moyo et al., 2016. After the shodhana of GR with Goghrita (clarified cow’s butter), all bands of GR were observed, along with new bands at 2,912 and 2,812 (CH_3_ and CH_2_ of fatty acids of Goghrita), 1,746 (C=O band of the carbonyl group), and 1,466 cm^−−1^ (–CH and =CH– bands of fatty acids of Goghrita) (Supplementary Figure S2). All bands of GR and SGR were observed in the FTIR spectrum of LSR, along with broad bands at approximately 3,380 (O–H stretching and H-bonded), 1,740 (C=O band of the carbonyl group), 1,635 (C=C stretching), 1,384, 1,151 (C–N stretching), and 1,030 cm^−1^ due to the presence of the organic metabolites of Shunthi (*Zingiber officinalis* Roscoe) and Nagawalli (*Piper betel* Linn) along with Goghrita (Figure 2). It has been reported than levigation allowed for the free binding sites to interact with metal ions, with consequent broadening taking place along with a band intensity decrease (Ebrahimian et al., 2023).

### 3.4 XRD analysis

**FIGURE 3:**
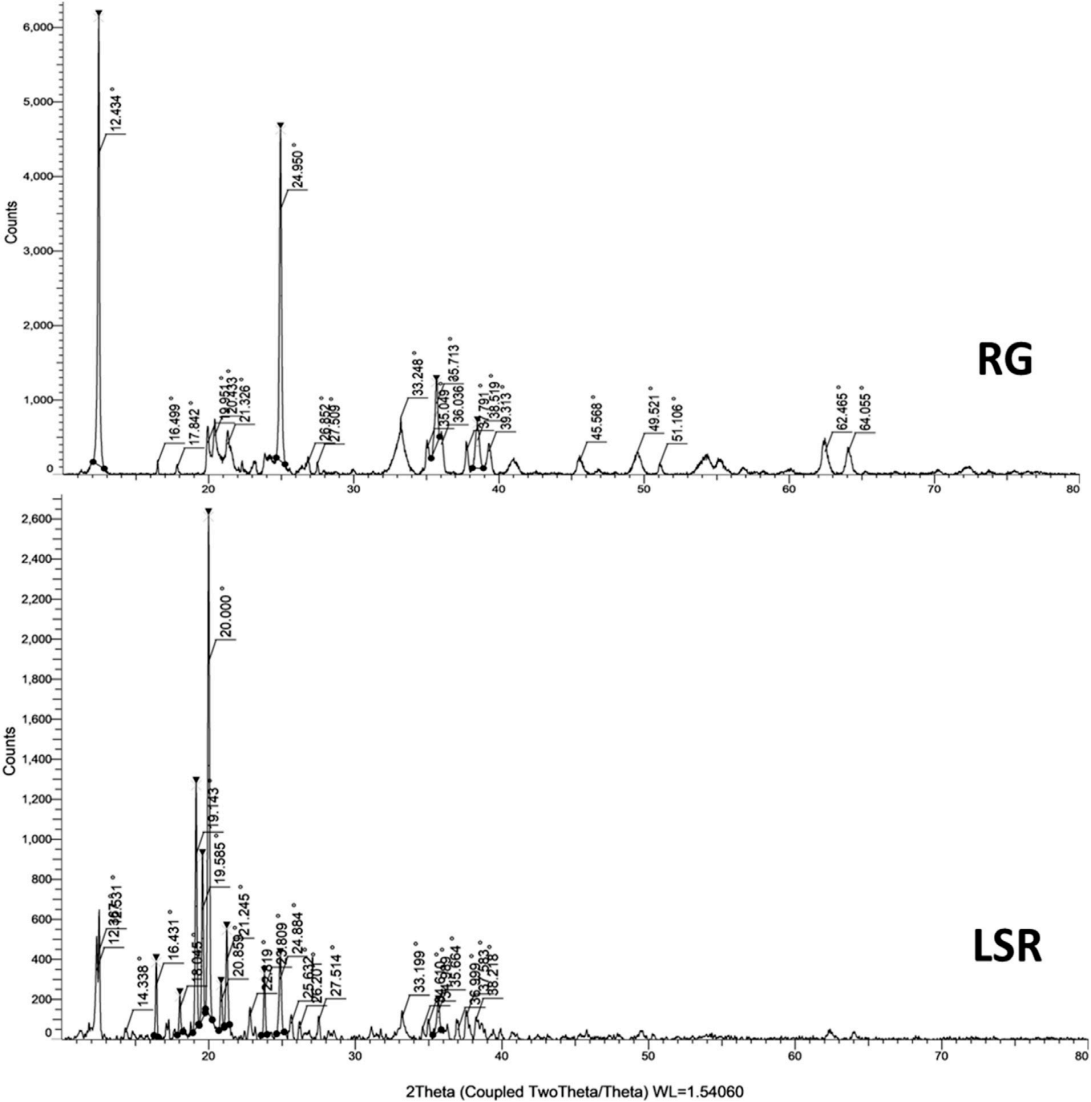
XRD analysis of raw RG and LSR.

Plant-based drugs, like herbo-mineral formulations, are most often amorphous in nature. The crystallinity of LSR along with raw RG and intermediate SRG was calculated using XRD analysis. 2Ɵ values were taken on the *X*-axis, and the corresponding intensities were taken on the *Y*-axis (Figure 3 and Supplementary Figure S2). Figure 3 shows the XRD pattern of RG with 20 diffraction peaks at ∼12°, ∼16°, ∼17°, ∼19°, ∼20°, ∼21°, ∼24°, ∼26°, ∼27°, ∼33°, ∼35°, ∼36°, ∼37°, ∼38°, ∼39°, ∼45°, ∼49°, ∼51°, ∼62°, and ∼64°. The three most intense peaks occurred at ∼12° (100%), ∼24° (82%), and ∼35° (22%). Similar peaks were observed in SRG, with a relative intensity increase at ∼20°, ∼35°, ∼36°, ∼62°, and ∼64°. In comparison with XRD patterns of RG with SRG, three peaks at ∼16°, ∼17°, and ∼19° were not observed in the SRG sample, which showed the conversion of goethite and hematite under heating (Capel et al., 2006). In comparison to RG and SRG with LSR, drastic changes were observed in the XRD patterns of LSR. A total of 24 diffraction peaks at ∼12°, ∼14°, 16°, ∼18°, ∼19°, ∼20°, ∼21°, ∼22°, ∼23°, ∼24°, ∼25°, ∼26°, ∼27°, ∼33°, ∼34°, ∼35°, ∼36°, ∼37°, ∼38°, ∼49°, ∼62°, and ∼64°, with the four most intense peaks occurring at 12° (23%), ∼19° (34%), 20° (100%), and ∼21° (20%), were observed. The omission of the selected peaks in LSR and the additional new peaks were due to the application of the Shunthi fine powder in the LSR formulation (Guglielmi et al., 2022; Teklay et al., 2023). The crystallinity of RG, SRG, and LSR compounds based on XRD analysis showed a similarity with iron oxide (01-088-0434 (N)) with a rhombohedral lattice (Moyo et al., 2016; Teklay et al., 2023).

The X-ray diffraction pattern is shown in Figure 1. The peaks at 2θ values of ∼27°, ∼33°, ∼35°, 39°, ∼55°, and ∼67° correspond to ά-Fe_2_O_3_ having a hexagonal phase (space grouping R3c (167) with JCPDS file no. 33-0664) (Mukherjee et al., 2016). The broad and sharp peaks are indications of nanoparticles (particles with a small size) and macroparticles (particles with a large size), respectively. Most of the peaks in LSR were sharp, which indicates the formation of macroparticles due to the capping ability of phytochemicals of Shunthi (Ragupathi et al., 2014). Broad peaks were observed in RG, indicating the formation of nanoparticles, as confirmed in SEM-EDX analysis. The crystalline phase remained rhombohedral after heating. The XRD studies revealed that RG and SRG contained ferric oxide, aluminum silicate hydroxide, and Al_4_(OH)_8_(Si_4_O_10_) with triclinic lattices. The XRD studies revealed that LSR contained ferric oxide, aluminum silicate hydroxide, and Al_4_(OH)_8_(Si_4_O_10_) with a triclinic lattice, a SiO_2_ tetragonal lattice, and a monoclinic lattice of montmorillonite. The crystallinity of RG was not affected by heating and levigation, as RG was mildly heated with clarified butter followed by levigation with Nagawalli (*Piper betel* Linn.) (Figure 3 and Supplementary Figure S2). In comparison with the literature data, the peaks at ∼20°, ∼21°, ∼26°, ∼33°, ∼39°, ∼42°, and ∼45° were of hematite and goethite; the peaks at ∼16°, ∼17°, and ∼29° were of kaolinite; the peaks at ∼24°, ∼26°, ∼49°, ∼51°, ∼62°, and ∼64° were of quartz; and the peaks at ∼12°, ∼23°, ∼35°, ∼37°, and ∼38° were of illite (Moyo et al., 2016; Teklay et al., 2023).

Gairika has a red color due to the presence of hematite (Fe_2_O_3_), and the main peaks of hematite were observed at ∼33°, ∼39°, ∼42°, and ∼45° (Mortimore et al., 2004; Elias et al., 2006). All of these minerals (like hematite and goethite (α-FeO(OH)), kaolinite (Al₂Si₂O₅(OH)₄), quartz, and illite ((K,H_3_O)(Al,Mg,Fe)_2_(Si,Al)_4_O_10_[(OH)_2_·(H_2_O)])) were observed in the XRD analysis of RG, SRG, and LSR. The reported expected percentage of hematite, kaolinite, quartz, illite, and calcite in Gairika (red ochre) was 30.87%, 7.00%, 55.87%, 3.52%, and 2.70%, respectively (Elias et al., 2006). In the present study, in XRD analysis, as per the peak intensity, the expected percentage of hematite, kaolinite, quartz, illite, and calcite in Gairika matched with the experimental study.

### 3.5 SEM-EDX analysis

**FIGURE 4:**
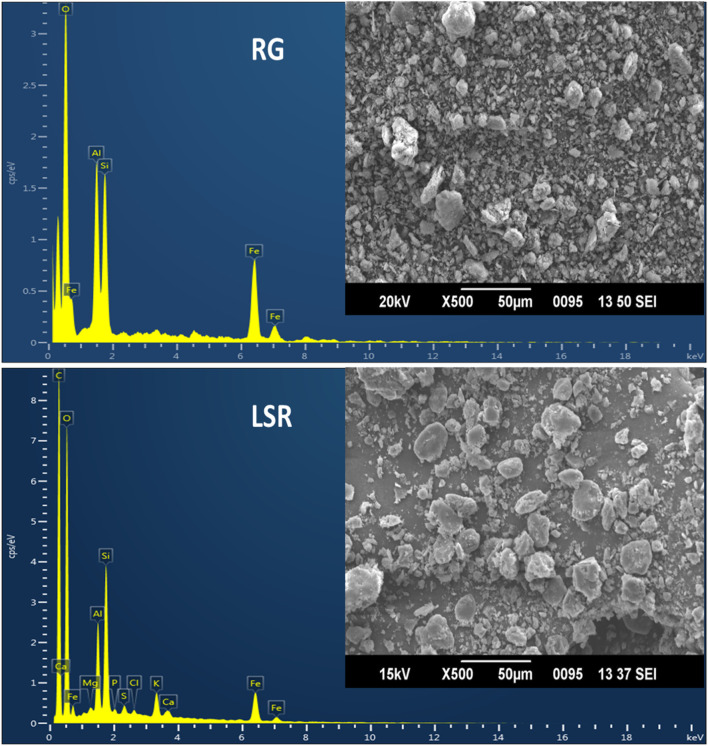
SEM-EDX analysis of raw RG and LSR.

In the SEM-EDX analysis of RG, the atomic percentage of O, Al, Si, and Fe was 60.49, 14.2, 13.88, and 11.43%, respectively. In the case of raw SRG, the atomic percentage of O, Al, Si, K, and Fe was 61.32, 14.81, 14.95, 0.21, and 8.31%, respectively. In the case of raw LSR, the atomic percentage of C, O, Mg, Al, Si, P, S, Cl, K, Ca, and Fe was 53.78, 37.98, 0.14, 2.14, 3.17, 0.12, 0.19, 0.11, 0.59, 0.14, and 1.65%, respectively. The overall percentage of Fe decreased from shodhana to the LSR preparation. As compared to RG, during the shodhana process, an approximate decrease of 27% in the Fe content was observed. Similarly, an approximate decrease of 85% in the Fe content was noticed for the conversion of RG into LSR. There was evidence of the formation of chelating complexes when GR was purified with Goghrita followed by levigation with Nagawalli (*Piper betel* Linn.). In the current study, the observed size of GR and SRG was 500 nm and 2,000 nm, respectively, which changed to 5–8 µm (in case of LSR) with the addition of Shunthi (*Zingiber officinalis* Roscoe) powder, followed by levigation with Nagawalli. Red-colored RG (500 nm size) changed into light-red SRG (2,000 nm size) when RG was purified using Goghrita. The atomic composition of SRG resembled that in a recent study by Guglielmi et al., 2022. The disc-shaped and rod-shaped particles observed in SEM-EDX analysis, shown in Figure 4, represented hematite and goethite (Mortimore et al., 2004). The SEM-EDX analysis of LSR revealed the formation of symmetric disc-shaped shaped macroparticles 5–8 µm in size (Figure 4). In the current study, the rod-shaped particles (goethite) converted into hematite with heating because of the loss of water molecules. In the intermediate sample, SRG was in between the images of RG and LSR, showing the conversion of goethite to hematite (Supplementary Figure S4).

### 3.6 Thermal analysis

**FIGURE 5:**
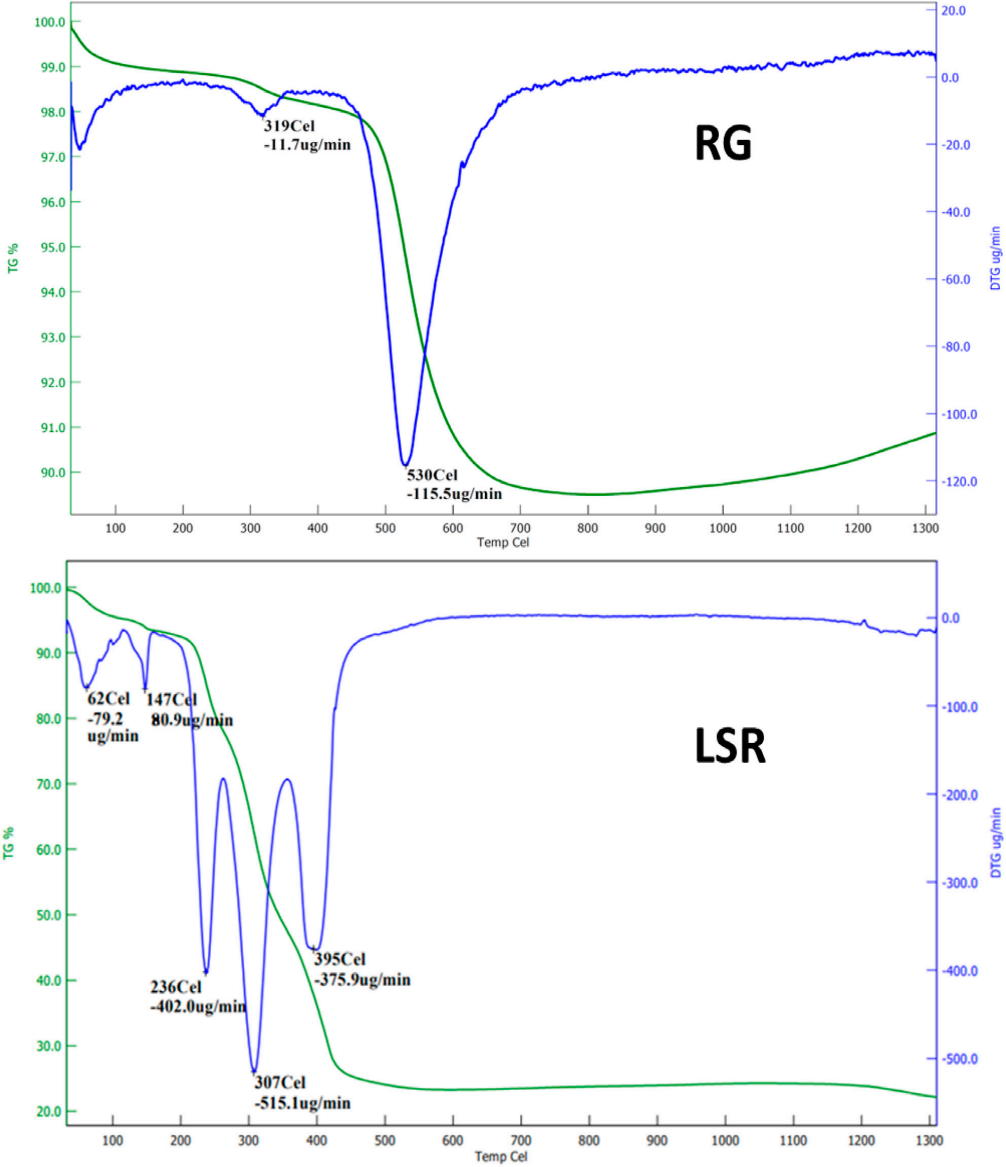
TGA analysis of raw RG and LSR.

The DTG graph of RG showed a loss of water below 100°C, with the initial sample decomposition of 11.7 μg/min at 319°C, and overall, the final sample degradation of 115.5 μg/min occurred at 530°C. In the TGA thermogram, RG or raw *Gairika* showed a 2% loss at approximately 319°C and a loss of almost 11% w/w at approximately 630°C (Figure 4). The DTG graph of SRG showed a loss of water below 100°C, initially 411.2 μg/min at 320°C, 173.1 μg/min at 390°C, and 167.1 μg/min at 530°C. In the TGA thermogram of SRG, a 2% loss at approximately 305°C and a 28% loss at approximately 594°C were observed (Supplementary Figure S5). The DTG graph of LSR showed a loss of water below 100°C, initially 78.8 μg/min at 62°C, 80.9 μg/min at 147.0°C, 402.0 μg/min at 236°C, 515.1 μg/min at 307°C, and 375.9 μg/min at 397°C. In the TGA thermogram of LSR, a 5% loss at approximately 150°C, 83% loss at approximately 237°C, and 73% loss at approximately 471°C were observed (Figure 5). Thermal analysis confirmed the fact that RG was converted into SRG by interactions of Goghrita (clarified cow’s butter) with RG. The maximum amount of Goghrita (clarified cow’s butter) was lost in the SRG thermal analysis at 320°C (Supplementary Figure S5). In the final formulation of LSR, there were interactions of Goghrita (clarified cow’s butter) and herbs (Shunthi and Nagawalli); consequently, more sample loss occurred before 320°C (Figure 4). In the thermal analysis of LSR, a three-step sample loss was observed at 236, 307, and 395°C. The maximum sample loss occurred at 307°C followed by 236°C due to the loss of Goghrita (clarified cow’s butter), and phytochemicals of herbs (Shunthi and Nagawalli) formed a complex with RG or SRG (Guglielmi et al., 2022).

### 3.7 GC–MS/MS analysis

**FIGURE 6:**
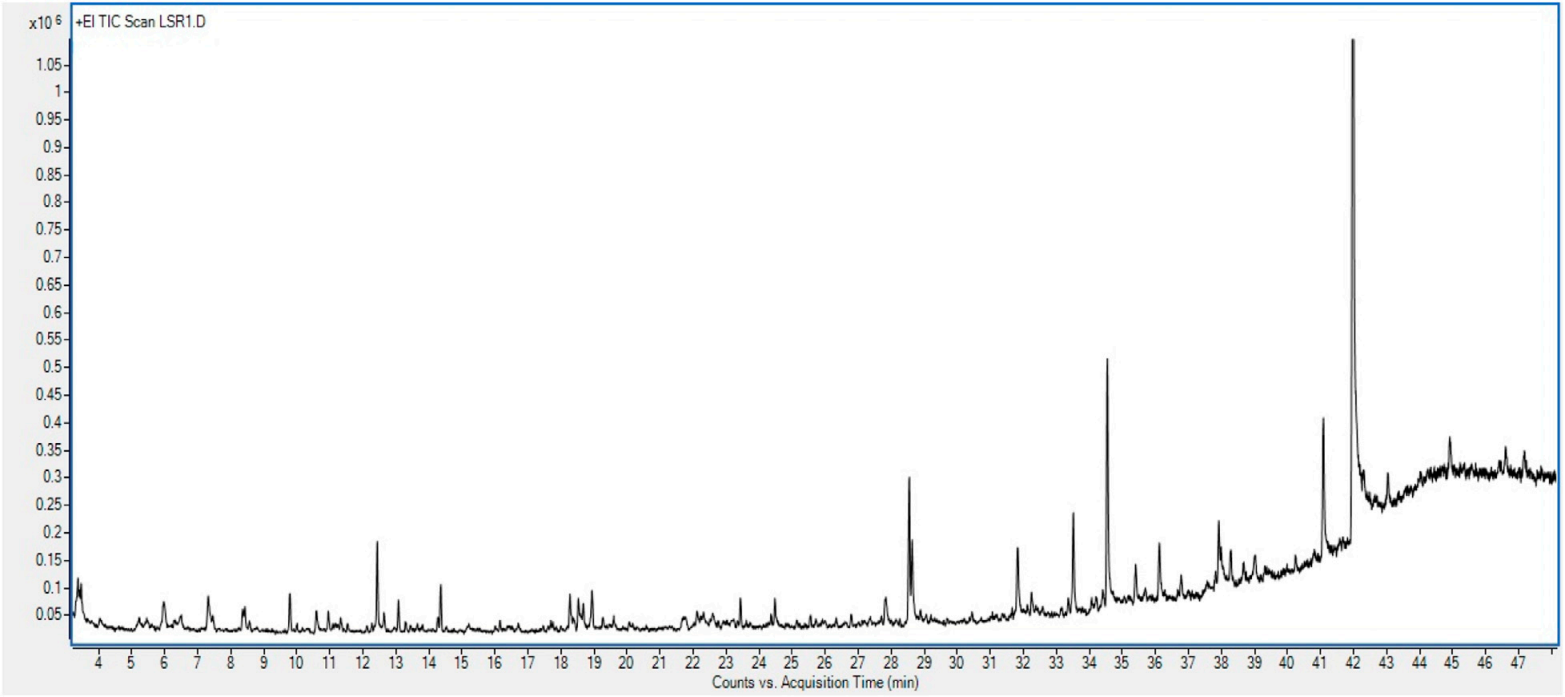
GC–MS chromatogram of the methanolic extract of LSR tablets.

GC–MS/MS analysis was carried out to check the presence of volatile components in Goghrita (clarified cow’s butter) and in phytochemicals of herbs (Shunthi and Nagawalli). The volatile components of LSR are mentioned in Table 2 and Figure 6. A total of 22 compounds were identified from the library search. The components of butter ((Z)-13-docosenamide) showed the highest percentage content along with palmitic acid, oleic acid, stearic acid, m-cymene, and 1-methyldodecyl methoxyacetate. The volatile compounds of Shunthi ([6]-gingerone, [6]-shogaol, [8]-shogaol, [10]-gingerol, and [10]-shogaol) and Nagawalli (betulin, myristic acid, and m-cymene) were also identified.

**TABLE 2:** List of major phytochemicals analyzed by GC–MS/MS with their RTs (in minutes).

RT (in min)	Phytochemical	% peak area
7.314	2-Ethyl-1-decanol	1.97
10.593	3-(1-Benzyl-1H-imidazol-2-yl)-5-methylisoxazole	0.78
10.950	1-Ethyl dodecyl methoxyacetate	0.54
12.443	m-Cymene	2.62
13.071	Nonadecane	0.75
18.272	2-Undecanone	1.35
18.519	1-Methyl dodecyl methoxyacetate	1.46
18.934	2,4-di-t-Butylphenol	1.40
23.426	11-(1-Ethylpropyl) heneicosane	0.72
24.469	Myristic acid	1.07
27.835	tert-Hexadecanethiol	1.44
28.545	Palmitic acid	4.83
28.627	Butyl octyl phthalate	3.17
31.825	Oleic acid	3.19
32.245	Stearic acid	0.61
33.515	[6]-Gingerone	0.48
34.539	[6]-Shogaol	3.85
35.399	Zingerone	1.38
36.114	[6]-Gingerol	2.16
37.916	[8]-Shogaol	2.19
38.283	Betulin	1.39
41.085	[10]-Shogaol	6.19
41.903	(Z)-13-docosenamide	47.54

### 3.8 Antioxidant and antacid activities

**FIGURE 7:**
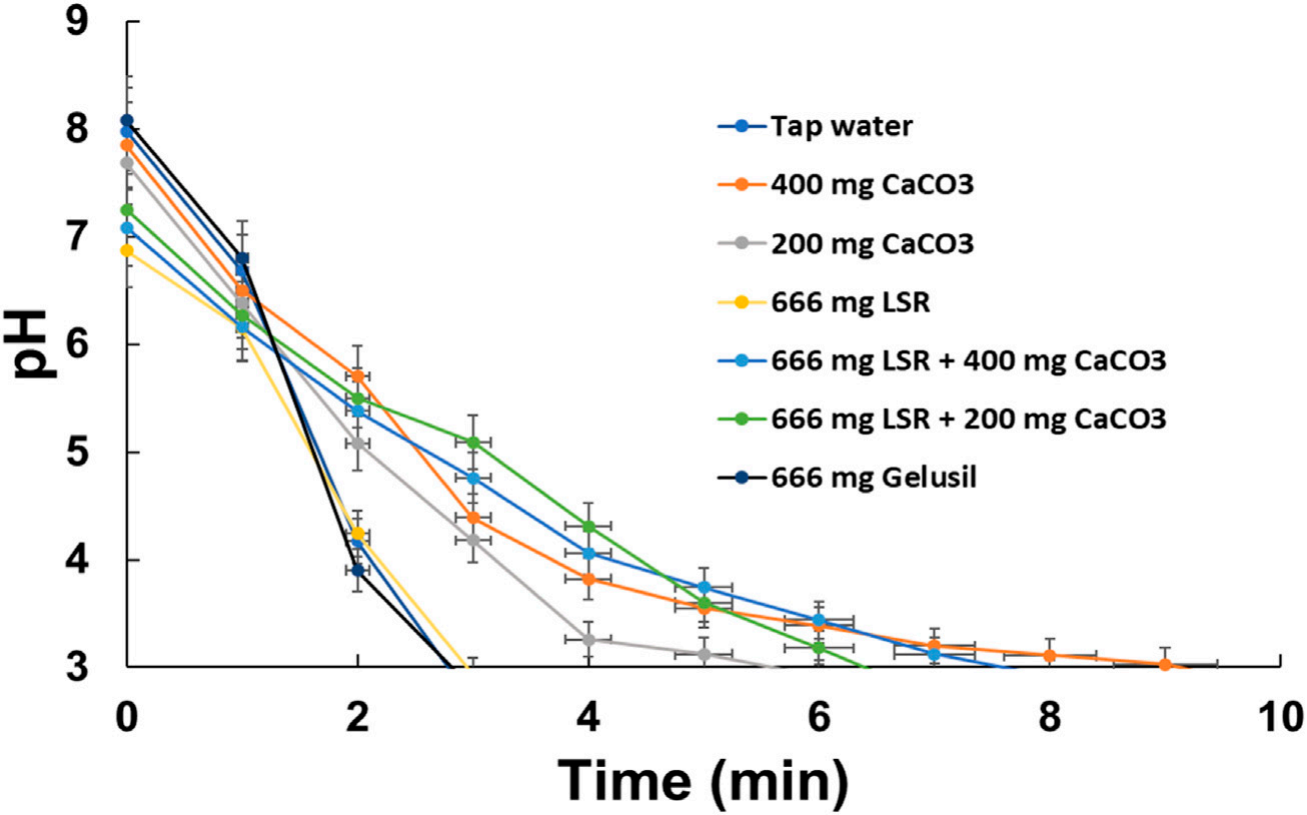
Antacid activity of LSR.

The DPPH assay revealed an IC_50_ value of 12.16 ± 1.23 mg/mL, which was 0.0029 for TEAC. The phytochemicals Shunthi and Nagawalli have a wide range of medicinal applications, including antioxidant properties. This is the first report on the antioxidant activities and antacid activity of LSR. The antacid activity of LSR along with Shunthi, a standard (CaCO_3_), and the market drug Gelusil is depicted in Figure 7. It has been observed that 666 mg of the LSR formulation in combination with 400 mg of the standard CaCO_3_ has an antacid activity (9.8 ± 0.17 min) close to that of the 400 mg standard (CaCO_3_) (11.2 ± 0.35 min). Even 666 mg of LSR alone showed an antacid activity (6.2 ± 0.15 min) higher than that of 666 mg of the market drug Gelusil (4.5 ± 0.17 min), which demonstrated the antacid potency of LSR. The antacid activity of the major raw drug (Shunthi) of LSR was observed to be 8.2 ± 0.13 min. The antacid activity of the LSR combination (i.e., a 1:1:1 combination of Nagawalli:RG:400-mg standard of CaCO_3_) was equivalent to that of LSR alone (6.2 ± 0.14 min). From the aforementioned observations, it was clear that LSR in combination with 400 mg of the standard CaCO_3_ is a potent antacid drug. *In vitro* studies revealed that Shunthi plays a main role in antacid potency, followed by the presence of Al, Ca, and Mg in LSR, as confirmed by FTIR, XRD, and SEM-EDX analysis.

There are limited reports on herbal and herbo-mineral formulations with antacid potential. In herbal drug development, dose calculation is a major problem, whereas in the current study, the dose (666 mg or 400 mg) was decided as per the report of Christensen et al. (2015). Antacids are used for gastroesophageal reflux-related symptoms, and the FDA recommends them as the first-line treatment for heartburn in pregnancy (Garg et al., 2022). Roopa et al., 2010 reported that triphala powder (contained in the *Terminalia chebula* fruit) exhibited antacid activity (Roopa et al., 2010). It has been reported that cold milk and broccoli present antacid activity, which was comparable to that of ENO and sodium bicarbonate (Panda et al., 2017). The antacid activity of milk and broccoli was correlated with the amount of Ca and the antioxidant activity (Panda et al., 2017). In the present study, the LSR formulation is rich in Ca (Table 1), which may be a reason for the significant antacid activity of LSR. Table 1 depicts that the LSR formulation contained a significant content of essential metal ions, including Fe, Mg, Al, K, and Na. All of these metal ions can play an important role in nutrition by enhancing the antioxidant potential (Kumar et al., 2019d). Under an artificial stomach model, the combination of CaCO_3_ and L*. aestuans* showed significant antacid activity (i.e., >CaCO_3_), and the same trend was observed in the present study.

L*. aestuans* is classically defined as an antacid herb in Ghana (Christensen et al., 2015). The methanolic extract of the root of *Tephrosia purpurea* (L.) Pers shows moderate antacid activity (Sandhya et al., 2012). The aqueous extracts of the fruit rind of Garcinia indica show antacid activity which is less than that of CaCO_3_ (Panda and Khambat, 2013). In modern treatment, there are various antacids like Al-based (a high content of Al can cause constipation), Mg-based (can cause diarrhea), and NaCO_3_-based antacids (can cause Na alterations, which cause cardiovascular diseases and high amounts of CO_2_), but all of them present significant risks (Sandhya et al., 2012; Panda and Khambat, 2013; Christensen et al., 2015; Garg et al., 2022). In the present study, no metal, like Al, Mg, Na, and Ca, was added externally to prepare the herbo-mineral drug LSR, which has advantages over modern metal-based antacids. Shunthi and Nagvel were used to make LSR more nutrient-rich and health-oriented due to the presence of various phytochemicals, like polyphenols and flavonoids. Shunthi and Nagawalli are widely used traditional herbs in the Indian medicinal system, with a wide range of medicinal applications, showing the advantages of LSR over modern antacids.

### 3.9 Antimicrobial and prebiotic activities

**FIGURE 8:**
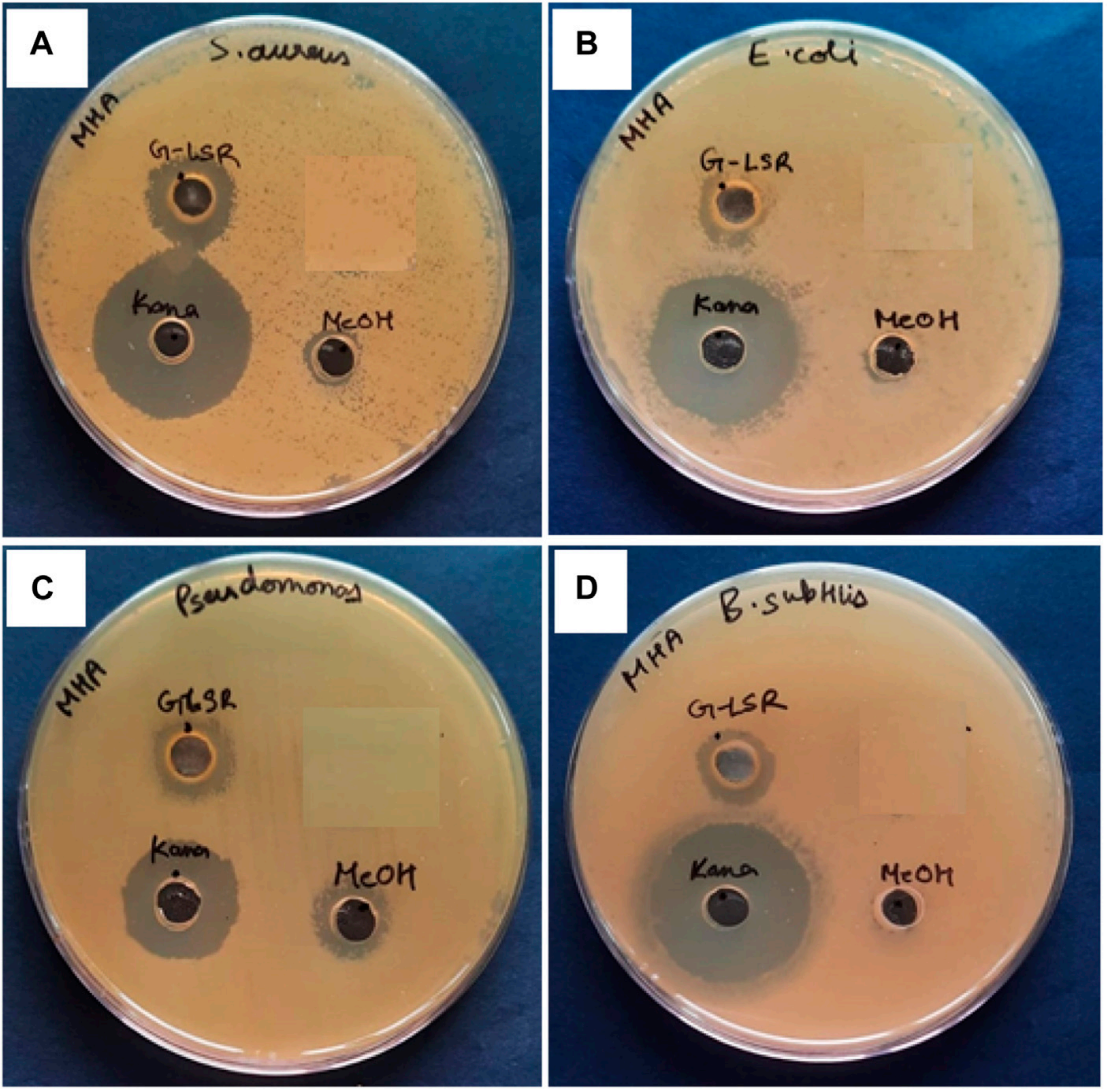
Antibacterial activity of 100 mg/mL of the LSR methanolic extract against **(A)** *Staphylococcus aureus* (MTCC no. 1430), **(B)** *Escherichia coli* (MTCC no. 1885), **(C)** *Pseudomonas aeruginosa* (MTCC no. 424), and **(D)** *Bacillus subtilis* (MTCC no. 619).

**FIGURE 9:**
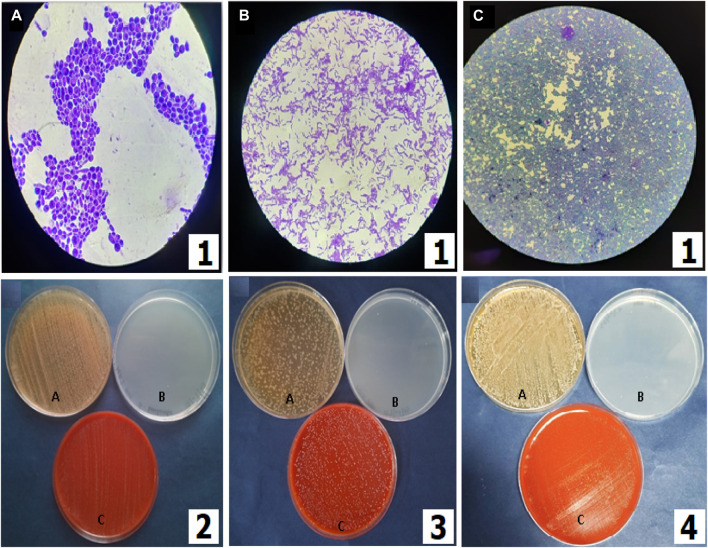
Prebiotic activities of LSR. (1) Microscopic visualization of **(A)** *Saccharomyces boulardii*, **(B)** *Lactobacillus paracasei* (MCC no. 4490), and **(C)** *Lactobacillus plantarum* (MTCC no. 1407) (100×). (2) *Saccharomyces boulardii* grown on G-LSR, (3) *Lactobacillus paracasei* (MCC no. 4490) grown on G-LSR, and (4) *Lactobacillus plantarum* (MTCC no. 1407) grown on G-LSR (for 2–4, A: MRS with dextrose as the complete media or positive control; B: MRS without dextrose as the minimal media or negative control; and C: MRS with the sample powder for the test).

The methanolic extract (100 mg/mL) of LSR showed 18 ± 4 mm zones of inhibition against bacterial stains *S*. *aureus*, *E. coli*, *P. aeruginosa*, and *B*. *subtilis* (Figure 8). There is no report on LSR yet, and its inhibition might be due to the presence of nano-sized red oxide along with fatty acids (of Goghrita) and phytochemicals of Shunthi (*Zingiber officinale*) and Nagawalli (*Piper betel* Linn.). In prebiotic studies, the gut microbiota-supported microorganisms, namely, *Saccharomyces boulardii*, *Lactobacillus paracasei*, and *Lactobacillus plantarum*, have shown prominent growth on LSR (Figure 9). The inhibition of pathogens (*S*. *aureus*, *E. coli*, *P. aeruginosa*, and *B*. *subtilis*) through LSR formulation and the growth of gut microbiota-supported strains (*S. boulardii*, *L*. *paracasei*, and *L. plantarum*) on the LSR formulation indicate the application of LSR as a prebiotic. There are selected studies on herbs and medicinal plants for prebiotic applications. Polyphenols of plants and herbs have shown strong correlations with prebiotic activities (Lu et al., 2017). Medicinal plants and their formulations, like *G. glabra*, U. rubra, and triphala formulations, have shown prebiotic results when tested against bacterial stains, like *Bifidobacterium spp*., *Lactobacillus spp*., and *Bacteroides spp*. (Peterson et al., 2018). The prebiotic action of herbs associated with the phytochemicals and fatty acids of herbs assist in the production of immune cells (Peterson et al., 2021). In a recent report by Peterson et al. (2018), Shunthi alone does not show any prebiotic activity. In the present study, Shunthi (*Zingiber officinale*) is present in LSR along with Goghrita and Nagawalli (*Piper betel* Linn.) leaf extracts. The presence of fatty acids (of Goghrita) and phytochemicals of Shunthi (*Zingiber officinale*) and Nagawalli (*Piper betel* Linn.) may have enhanced the prebiotic ability of LSR.

### 3.10 Probable mechanism of purification and levigation

**FIGURE 10:**
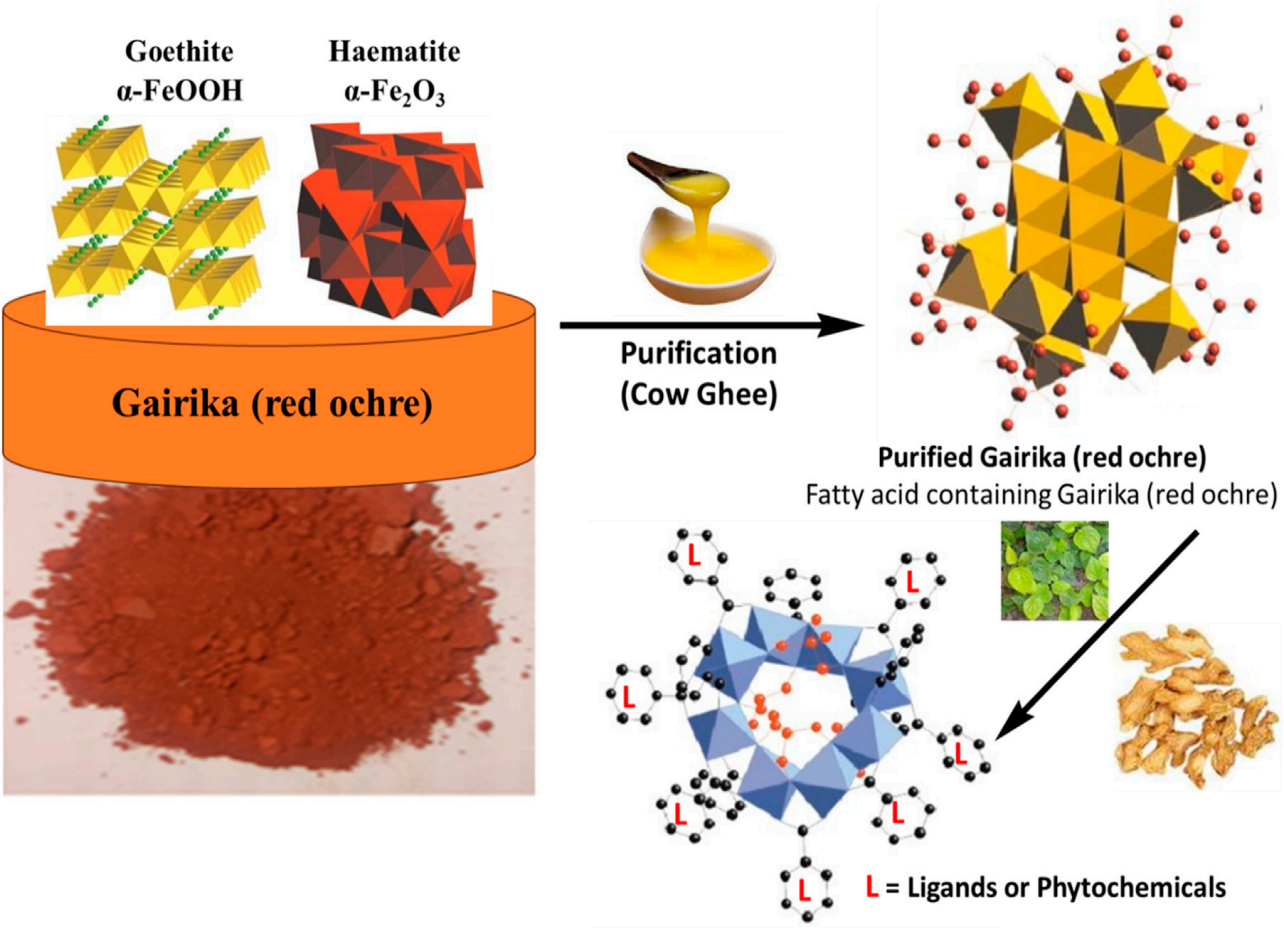
Probable mechanism of the formation of LSR from raw Gairika (red ochre).

LSR was prepared as per the classical literature for the sake of validation of the classical procedure. To validate the classical procedure, our first attempt was to understand the chemistry behind the pharmaceutical process. The overall mechanism for the formation of Laghu Sutashekhara Rasa from raw Gairika (red ochre) is depicted in Figure 10. During the purification process, fatty acids (red dots with bonds) of cow ghee were absorbed on raw Gairika (red ochre). A polycationic ferric species formation is reported in iron oxides, followed by the formation of polycationic corrugated planes of the oxyhydroxide phase. Such a transformation produces lath-shaped particles of Goethite (Jolivet et al., 2004). After the formation of fatty acids (of cow ghee) containing polycationic corrugated planes of Gairika (purified Gairika), the levigation process was employed where hydration took place due to the application of the water extract of Nagawalli leaves. During the levigation process, the phytochemicals of Shunthi and Nagawalli interacted with purified Gairika. The mode of interactions may be H-bonding between the phytochemicals and fatty acids, along with purified Gairika, and/or direct bonding of iron with chelating sites of the phytochemicals of Shunthi and Nagawalli. These interactions lead to the formation of macromolecules, that is, an enhancement in the size of molecules as compared to raw Gairika and purified Gairika, as depicted in Figure 4.

The reason behind the use of ghee in the shodhana of raw RG was to enhance the pH-dependent solubility of hydrophobic drugs. The RG has a weak basic property (pH 7–9) and should have a pH below the pKa value for better solubility and absorbability (Serajuddin, 2007). There are various hydrophobic formulations that have been prepared by the pH modification process. In the maximum pH modification process, lactic acid and oleic acid play the vital roles of enhancing solubility and bioavailability (Kalepu and Nekkanti, 2015; Lamsal et al., 2020). In the present study, cow ghee has been used because of the presence of fatty acids and lactic acid in it, as observed in the GC–MS study, so the purification of raw Gairika with cow ghee is an efficient technique for the enhancement of the solubility and bioavailability of Gairika, along with the detoxification of Gairika. It has been observed that the levigation process allows the hydration and co-crystallization mechanism, which enhances the shelf-life and bioavailability of drugs (Jolivet et al., 2004; Lu et al., 2019; Yoshimatsu et al., 1981). In the present study, purified Gairika is mixed with Shunthi and then levigated using the Nagawalli water extract, where Gairika oxides are hydrated and convert Fe_2_O_3_ into Fe_2_O_3_·nH_2_O. The mechanism of co-crystallization may take place between Fe_2_O_3_ and/or Fe_2_O_3_·nH_2_O and the phytoconstituents of Shunthi (gingerols or shogaols) and Nagawalli (rutin). In the present study, the pH of raw Gairika (8.4 ± 1.24) is decreased (LSR: 5.37 ± 0.65) with the levigation and purification process, which means that insoluble Fe(III) of Gairika is converted into soluble Fe(II). Overall, the purification of Gairika with cow ghee and levigation with Nagawalli may enhance the solubility, bioavailability, and shelf-life of LSR through the hydration and co-crystallization mechanism.

## 4 Conclusion

Here, the pharmaceutical validation of the Laghu Sutashekhara Rasa formulation was completed, and batch-to-batch reproducibility was achieved (Supplementary Material). The LSR formulation was characterized using advanced instrumentation, like HPTLC, FTIR, XRD, SEM-EDX, and TGA. This study could be used as a monograph or regulatory document after its incorporation in the Ayurvedic Pharmacopeia of India (API). The monograph, as per API or WHO, is mentioned in the Supplementary Material. The formulation of Laghu Sutashekhara Rasa showed significant antioxidant and antacid activities with CaCO_3_. The inhibition of pathogens (*S*. *aureus*, *E. coli*, *P. aeruginosa*, and *B*. *subtilis*) by the LSR formulation and the growth of gut microbiota-supported strains (*S. boulardii*, *L*. *paracasei*, and *L. plantarum*) on the LSR formulation were indicative for the application of LSR as a prebiotic. Furthermore, a detailed study is required to identify the neutralizing compound in the formulation and the mode of action. Moreover, toxicity studies should be carried out to check the formulation as either being an antacid phytomedicine or herbal medicine. The present study demonstrates the use of a traditional/Ayurvedic formulation as an antacid phytomedicine.

## Data Availability

The original contributions presented in the study are included in the article/Supplementary Material; further inquiries can be directed to the corresponding authors.

## References

[B1] Capel J. Huertas F. Pozzuoli A. Linares J. (2006). Red ochre decorations in Spanish Neolithic ceramics: a mineralogical and technological study. J. Archaeol. Sci. 33, 1157–1166. 10.1016/j.jas.2005.12.004

[B2] Carretero M. I. Gomes C. S. F. Tateo F., (2013). “Chapter 5.5 - clays, drugs, and human health,” in Faïza bergaya, gerhard lagaly, developments in clay science (Germany: Elsevier), 5, 711–764. 10.1016/B978-0-08-098259-5.00025-1

[B3] Chandra B. P. H. Prakash S. V. (2020). “Analysis of the Indian traditional loha shodhana process for biocompatibility,” in Saleem hashmi, imtiaz ahmed choudhury, encyclopedia of renewable and sustainable materials. Editor (Germany: Elsevier), 26–32. 10.1016/B978-0-12-803581-8.11266-4

[B4] Christensen C. B. Soelberg J. Jäger A. K. (2015). Antacid activity of laportea aestuans (L.) chew. /J. Ethnopharmacol. 171, 1–3. 10.1016/j.jep.2015.05.026 26023029

[B5] Duarte C. M. (2014). Red ochre and shells: clues to human evolution. Trends Ecol. Evol. 29, 560–565. 10.1016/j.tree.2014.08.002 25172406

[B6] Ebrahimian J. Khayatkashani M. Soltani N. Mohammed H. T. Tavakkoli N. Jafari M. (2023). Rosa Damascena mediated ZnO-Red Ochre nanocomposite for the electrochemical determination of 5-Fluorouracil. Arabian J. Chem. 16, 104586, 104586. 10.1016/j.arabjc.2023.104586

[B7] Elias M. Chartier C. Prévot G. Garay H. Vignaud C. (2006). The colour of ochres explained by their composition. Mater. Sci. Eng. B 127, 70–80. 10.1016/j.mseb.2005.09.061

[B8] Farooq S. Mehmood Z. Khan M. S. Ahmad I. (2019). “Chapter 22 - nanoparticles in ayurvedic medicine: potential and prospects,” in Mohd sajjad ahmad khan, iqbal ahmad, debprasad Chattopadhyay.New look to phytomedicine (China: Academic Press), 581–596. 10.1016/B978-0-12-814619-4.00023-9

[B9] Fordtran J. S. Morawski S. G. Richardson C. T. (1973). *In vivo* and *in vitro* evaluation of liquid antacids. N. Engl. J. Med. 1288, 923–928. 10.1056/nejm197305032881801 4693244

[B10] Garg V. Narang P. Taneja R. (2022). Antacids revisited: review on contemporary facts and relevance for self-management. J. Int. Med. Res. 50 (3), 030006052210864. 10.1177/03000605221086457 PMC8966100 35343261

[B11] Guglielmi V. Andreoli M. Comite V. Baroni A. Fermo P. (2022). The combined use of SEM-EDX, Raman, ATR-FTIR and visible reflectance techniques for the characterisation of Roman wall painting pigments from Monte d’Oro area (Rome): an insight into red, yellow and pink shades. Environ. Sci. Pollut. Res. 29, 29419–29437. 10.1007/s11356-021-15085-w PMC9001301 34196870

[B12] Hradil D. Hradilová J. Bezdička P. Serendan C. (2017). Late Gothic/early Renaissance gilding technology and the traditional poliment material “Armenian bole”: truly red clay, or rather bauxite? Appl. Clay Sci. 135, 271–281. 10.1016/j.clay.2016.10.004

[B13] Jones E. P. Knapp C. W. Venieri D. Christidis G. E. Elgy C. Valsami-Jones E. (2018). Greco-Roman mineral (litho)therapeutics and their relationship to their microbiome: the case of the red pigment miltos. J. Archaeol. Sci. Rep. 22, 179–192. 10.1016/j.jasrep.2018.07.017 30775415 PMC6360534

[B14] Kalepu S. Nekkanti V. (2015). Insoluble drug delivery strategies: review of recent advances and business prospects. Acta Pharm. Sin. B 5 (5), 442–453. 10.1016/j.apsb.2015.07.003 26579474 PMC4629443

[B15] Kotagasti T. (2015). Efficacy of Geru (red ochre) in controlling the bleeding in patients of Adolescent menorrhagia. CellMed 5, 12.1–12.3. 10.5667/tang.2015.0002

[B16] Krishnamachary B. Rajendran N. Pemiah B. Krishnaswamy S. Krishnan U. M. Sethuraman S. (2012). Scientific validation of the different purification steps involved in the preparation of an Indian Ayurvedic medicine, Lauha Bhasma. J. Ethnopharmacol. 142, 98–104. 10.1016/j.jep.2012.04.021 22561344

[B17] Kumar V. Kushwaha V. Charde V. Jagtap C. Gandhi Y. Grewal J. (2022a). The validated pharmaceutical standard operating procedure and quality control study of the coded polyherbal tablet formulation AYUSH SG-5. South Afr. J. Bot 151 (2022a), 319–327. 10.1016/j.sajb.2022.02.038

[B18] Kumar V. Kushwaha V. Gandhi Y. Mishra S. K. Charde V. Jagtap C. 2022b. A validated high-performance thin-layer chromatography method for the simultaneous quantification of 6-gingerol, guggulsterone E and guggulsterone Z in coded formulation AYUSH SG-5 prepared for rheumatoid arthritis. JPC-J Planar Chromat 35, 23–33. 10.1007/s00764-022-00153-9

[B19] Kumar V. Singh S. Singh A. Subhose V. Prakash O. (2019c). Assessment of heavy metal ions, essential metal ions, and antioxidant properties of the most common herbal drugs in Indian Ayurvedic hospital: for ensuring quality assurance of certain Ayurvedic drugs. Agric. Biotechnol. 18, 101018. 10.1016/j.bcab.2019.01.056

[B20] Kumar V. Singh S. Srivastava B. Bhadouria R. Singh R. (2019d). Green synthesis of silver nanoparticles using leaf extract of Holoptelea integrifolia and preliminary investigation of its antioxidant, anti-inflammatory, antidiabetic and antibacterial activities. J. Environ. Chem. Eng. 7, 103094. 10.1016/j.jece.2019.103094

[B21] Kumar V. Singh S. Srivastava B. Patial P. K. Kondalkar S. A. Bharthi V. (2019b). Volatile and semi-volatile compounds of Tephrosia purpurea and its medicinal activities: experimental and computational studies. Biocatal. Agric. Biotechnol. 20, 101222. 10.1016/j.bcab.2019.101222

[B22] Kumar V. Singh S. Srivastava B. Singh Sisodia B. Barthi B. Singh R. (2019a). High resolution GC/MS analysis of the Holoptelea integrifoli's leaves and their medicinal qualities. Biocatal. Agric. Biotechnol. 22, 101405. 10.1016/j.bcab.2019.101405

[B23] Kumar V. Singh S. B. Singh S. (2020). COVID-19: environment concern and impact of Indian medicinal system. J. Environ. Chem. Eng. 8, 104144. 10.1016/j.jece.2020.104144 33520648 PMC7836929

[B24] Lamsal B. Bhandari T. R. Panta P. Saiter J. M. Pokhrel S. Katuwal T. B. (2020). Preparation and physicochemical characterization of ghee and murcchita ghŗta. J. Ayurveda Integr. Med. 11, 256–260. 10.1016/j.jaim.2020.06.004 32798193 PMC7528008

[B25] Li X. Wu L. Wu R. Sun M. Fu Ke Kuang T. (2022). Comparison of medicinal preparations of Ayurveda in India and five traditional medicines in China. J. Ethnopharmacol. 284, 114775, 114775. 10.1016/j.jep.2021.114775 34742863

[B26] Lu Q. Dun J. Chen J.-M. Liu S. Sun C. C. (2019). Improving solid-state properties of berberine chloride through forming a salt cocrystal with citric acid. Int. J. Pharm. 554, 14–20. 10.1016/j.ijpharm.2018.10.062 30385378

[B27] Lu Q. Y. Summanen P. H. Lee R. P. Huang J. Henning S. M. Heber D. (2017). Prebiotic potential and chemical composition of seven culinary spice extracts. J. Food Sci. 82 (8), 1807–1813. 10.1111/1750-3841.13792 28678344 PMC5600121

[B28] Mortimore J. L. Marshall L.-J. R. Almond M. J. Hollins P. Matthews W. (2004). Analysis of red and yellow ochre samples from Clearwell Caves and Çatalhöyük by vibrational spectroscopy and other techniques. Spectrochimica Acta Part A Mol. Biomol. Spectrosc. 60, 1179–1188. 10.1016/j.saa.2003.08.002 15084337

[B29] Moyo S. Mphuthi D. Cukrowska E. Henshilwood C. S. van Niekerk K. Chimuka L. (2016). Blombos cave: middle stone age ochre differentiation through FTIR, ICP OES, ED XRF and XRD. Quat. Int. 404, 20–29. 10.1016/j.quaint.2015.09.041

[B30] Mukherjee D. Ghosh S. Majumdar S. Annapurna K. (2016). Green synthesis of α-Fe 2 O 3 nanoparticles for arsenic(V) remediation with a novel aspect for sludge management. J. Environ. Chem. Eng. 4, 639–650. 10.1016/j.jece.2015.12.010

[B31] Panda V. Khambat P. (2013). Evaluation of antacid activity of Garcinia indica fruit rind by a modified artificial stomach model. Bull. Env. Pharmacol. Life Sci. 2, 38–42. Available at: https://bepls.com/beplsjune2013/8.pdf.

[B32] Panda V. Shinde P. Deora J. Gupta P. (2017). A comparative study of the antacid effect of some commonly consumed foods for hyperacidity in an artificial stomach model. Complementary Ther. Med. 34, 111–115. 10.1016/j.ctim.2017.08.002 28917362

[B33] Pandit S. K Biswas T. K Debnath P. V Saha A. Chowdhury U. P Shaw B. (1999). Chemical and pharmacological evaluation of different ayurvedic preparations of iron. J. Ethnopharmacol. 65, 149–156. 10.1016/S0378-8741(99)00003-3 10465655

[B34] Peterson C. T. Iablokov S. N. Uchitel S. Chopra D. Perez-Santiago J. Rodionov D. A. (2021). Community metabolic interactions, vitamin production and prebiotic potential of medicinal herbs used for immunomodulation. Front. Genet. 12, 584197, 584197. 10.3389/fgene.2021.584197 33613632 PMC7886795

[B35] Peterson C. T. Sharma V. Uchitel S. Denniston K. Chopra D. Mills P. J. (2018). Prebiotic potential of herbal medicines used in digestive health and disease. J. Altern. Complement. Med. 4, 656–665. 10.1089/acm.2017.0422 PMC6065514 29565634

[B36] Ragupathi C. John Kennedy L. Judith J. (2014). A new approach: synthesis, characterization and optical studies of nano-zinc aluminate. Adv. Powder Technol. 25, 267–273. 10.1016/j.apt.2013.04.013

[B37] Ribeiro T. B. Costa C. M. Lopes T. B. Silva S. Veiga M. Monforte A. R. 2021 Prebiotic effects of olive pomace powders in the gut: *in vitro* evaluation of the inhibition of adhesion of pathogens, prebiotic and antioxidant effects. Food Hydrocoll. 112, 2021, 106312. 10.1016/j.foodhyd.2020.106312

[B38] Roopa G. Bhat R. S. Dakshina M. S. (2010). Formulation and evaluation of an antacid and anti-ulcer suspension containing herbal drugs. Biomed. Pharmacol. J. 3 (1), 01–06.

[B39] Sandhya S. Venkata R. K. Vinod K. R. Chaitanya R. (2012). Assessment of *in vitro* antacid activity of different root extracts of Tephrosia purpurea (L.) Pers by modified artificial stomach model. Asian pac. J. Trop. Biomed. 2, 1487–1492. 10.1016/S2221-1691(12)60442-0

[B40] Serajuddin A. T. (2007). Salt formation to improve drug solubility. Adv. Drug Deliv. Rev. 59 (7), 603–616. 10.1016/j.addr.2007.05.010 17619064

[B41] Shweta M. Shivshankar R. Galib D. B. (2020). Shelf life evaluation of Laghu Sutashekhara Rasa – a preliminary assessment. J. Ayurveda Integr. Med. 11, 213–216. 10.1016/j.jaim.2018.01.007 30638717 PMC7527809

[B42] Teklay M. Thole J. T. Ndumbu N. Vries J. Mezger K. (2023). Mineralogical and chemical characterization of ochres used by the Himba and Nama people of Namibia. J. Archaeol. Sci. Rep. 47, 103690, 103690. 10.1016/j.jasrep.2022.103690

[B43] Yoshimatsu K. Nakabayashi S. Ogaki J. Kimura M. Horikoshi I. (1981). Dielectric study on the water of crystallization of berberine chloride. Yakugaku Zasshi J. Pharm. Soc. Jpn. 101 (12), 1143–1148. 10.1248/yakushi1947.101.12_1143 7338802

