# Supplementary material for: Establishment of the mechanism of purification and levigation of green chemistry-assisted biocomposites of red ochre (Gairika): synthesis, characterization, and antibacterial, prebiotic, antioxidant, and antacid activities of the traditional Ayurvedic medicine Laghu Sutashekhara Rasa
Source: Front Chem. 2023 Nov 23;11:1271157. doi: 10.3389/fchem.2023.1271157 (PMC10701397; doi:10.3389/fchem.2023.1271157)
Supplement: Supplementary file 1 [file DataSheet1.docx]

**Supplementary Data**


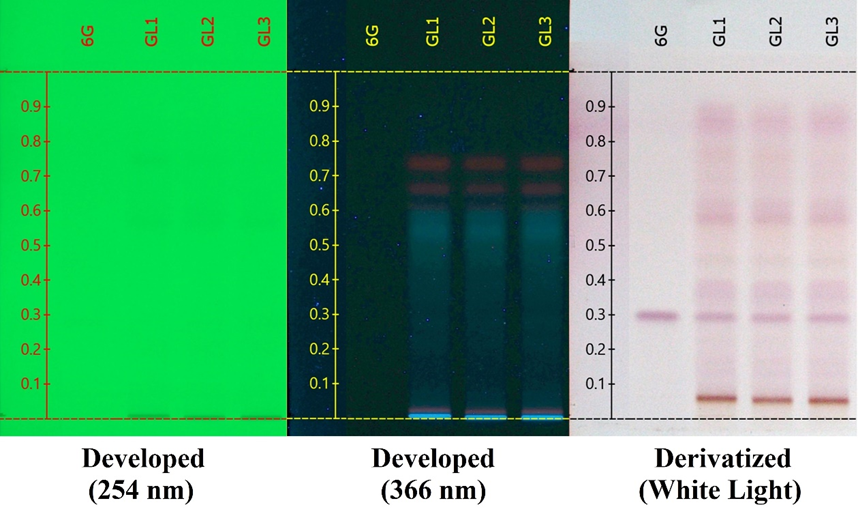


**Figure.** HPTLC analysis of LSR.


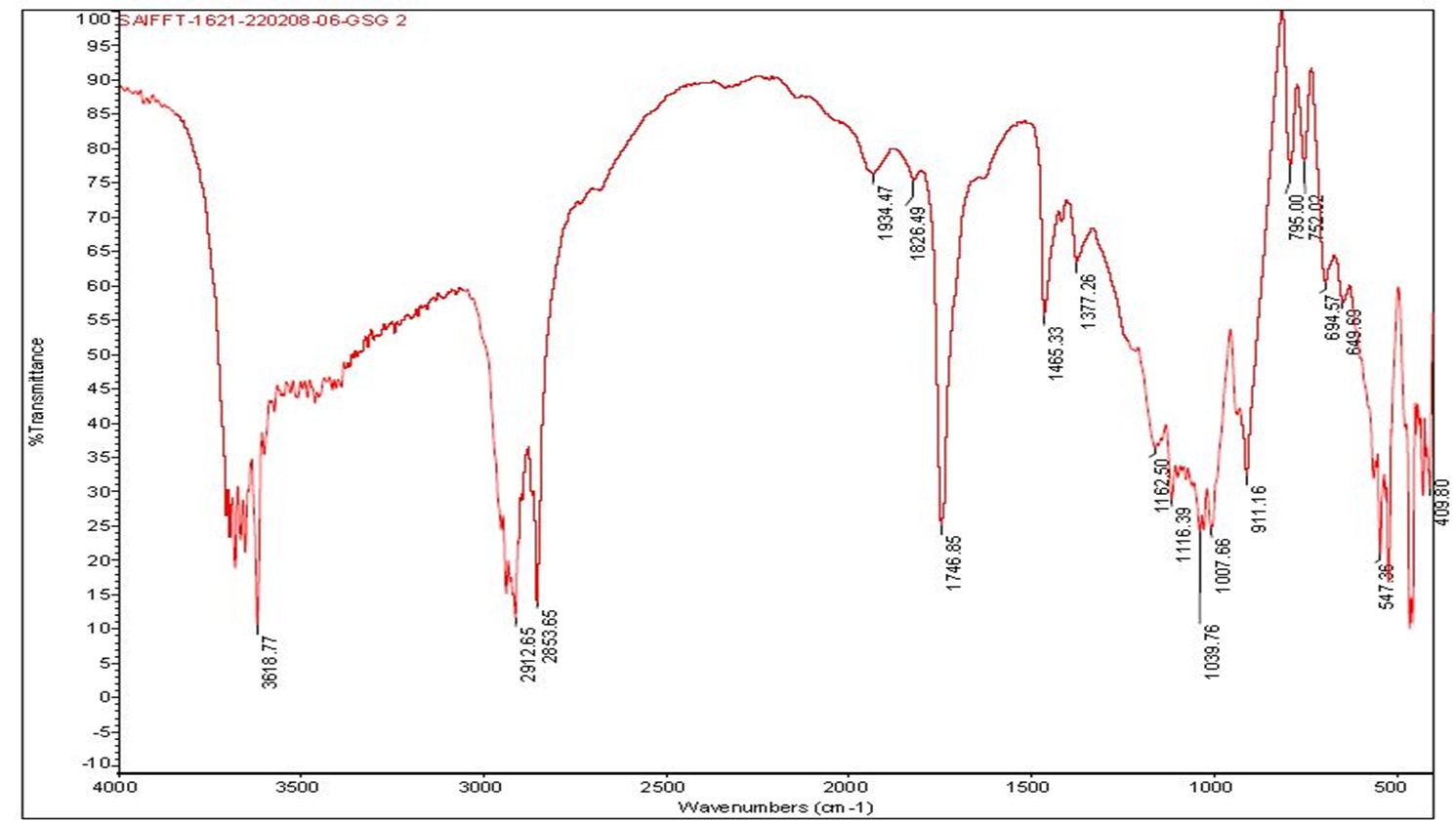


**Figure.** FTIR Analysis of shodita (purified) Gairika (SRG).


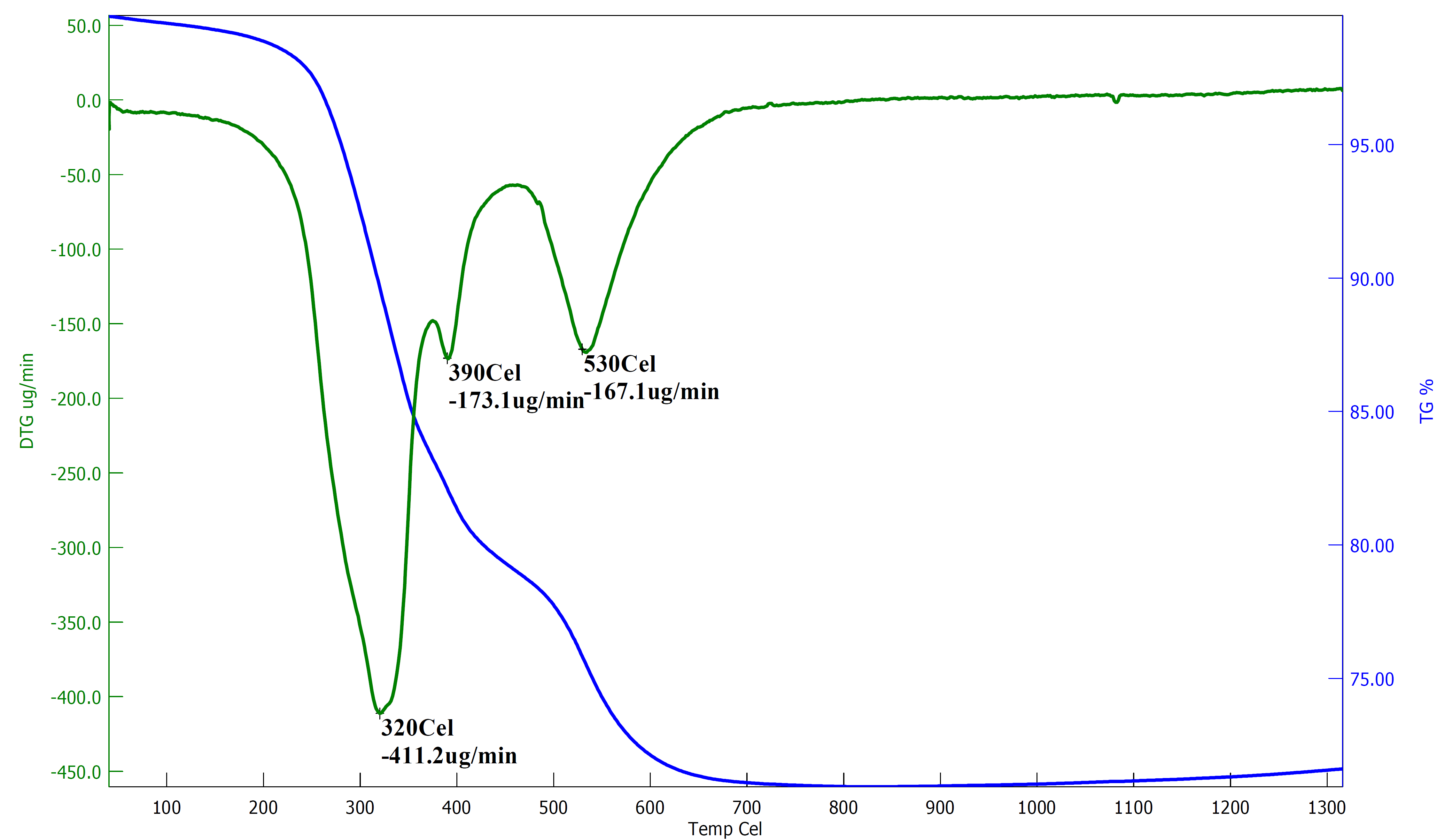


**Figure.** TGA Analysis of shodhita (purified) Gairika (SRG).


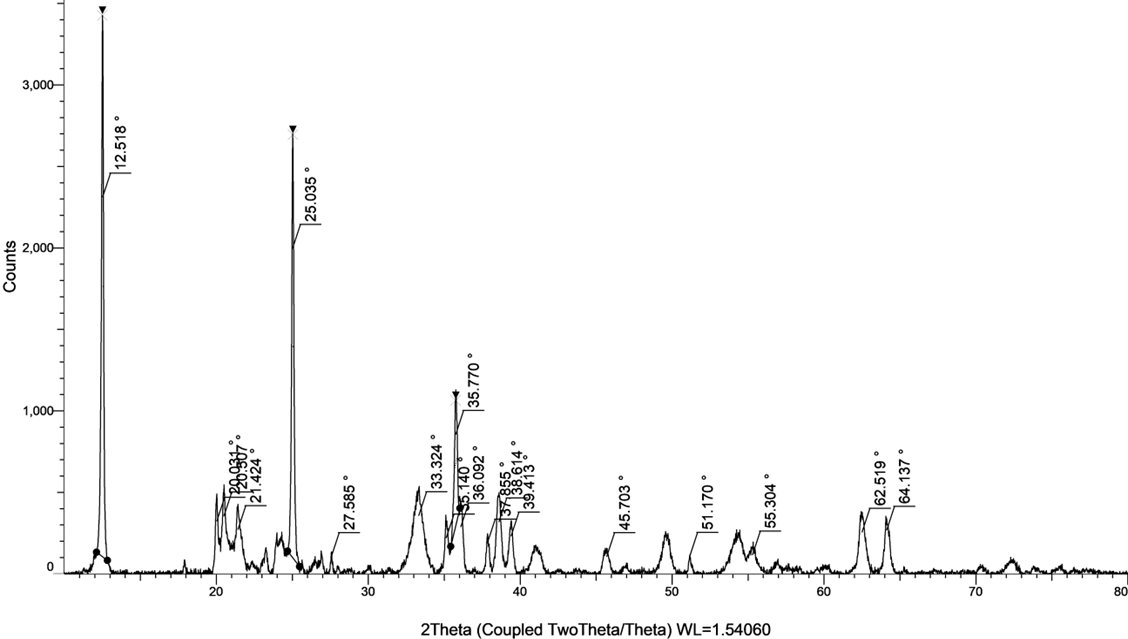


**Figure.** XRD Analysis of shodita (purified) Gairika (SRG).


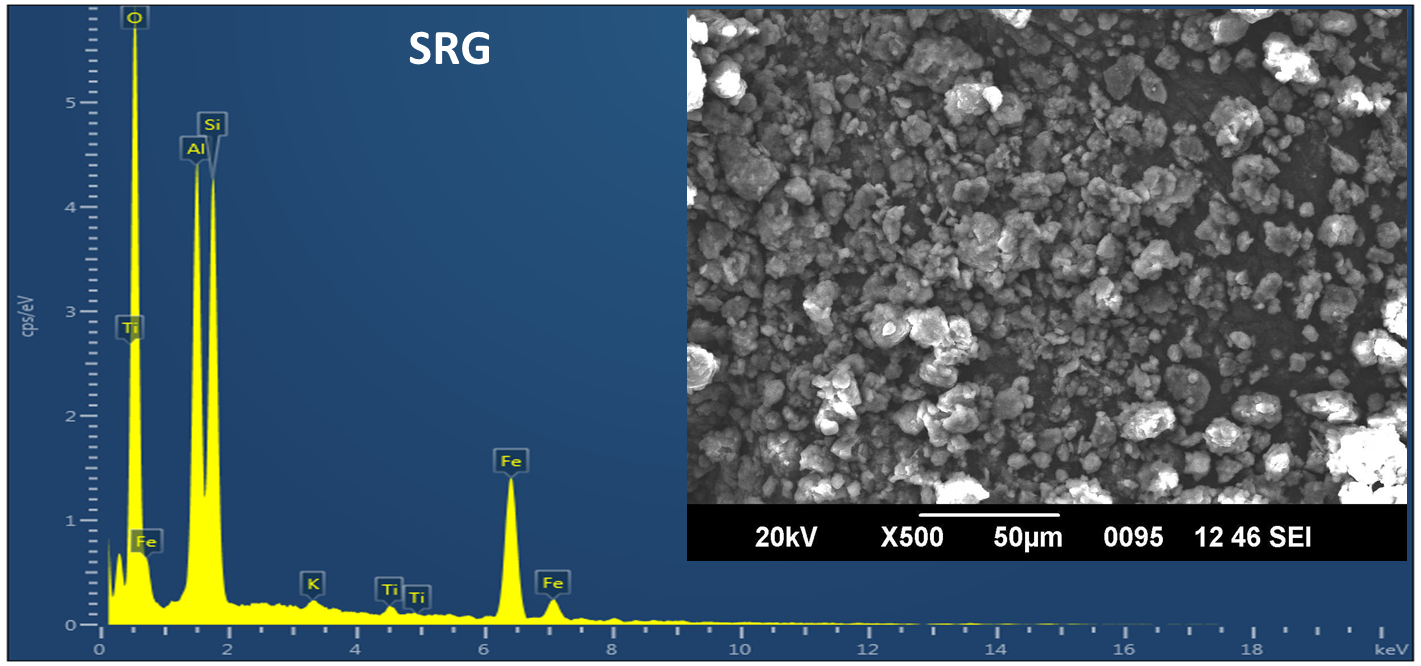


**Figure.** SEM-EDX Analysis of shodita (purified) Gairika (SRG).

**Shunthi (*Zingiber Officinalis* Roscoe)**

1. **A. Organoleptic characters** Shunthi (*Zingiber Officinalis* Roscoe)**:**

| Characteristic/Name of the Raw Drug | Shunthi (*Zingiber Officinalis* Roscoe) |
| --- | --- |
| Colour | Creamish yellow |
| Odour | Aromatic |
| Taste | Pungent |
| Texture | Fibrous |

1. **Powder microscopy**

Powder microscopy of sample shows presence of fragments of cork cells in surface view and transverse view, oleo-resin content in parenchyma cells, simple starch grains in abundance, xylem vessels and septate fibres. All the observed characters comply with the Ayurvedic Pharmacopoeia of India.

| 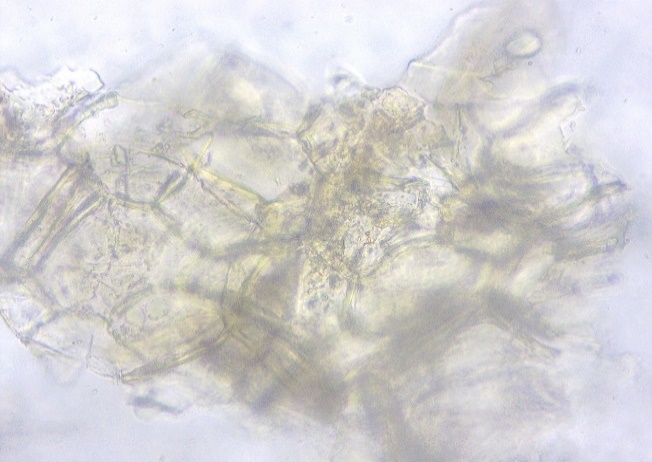 | 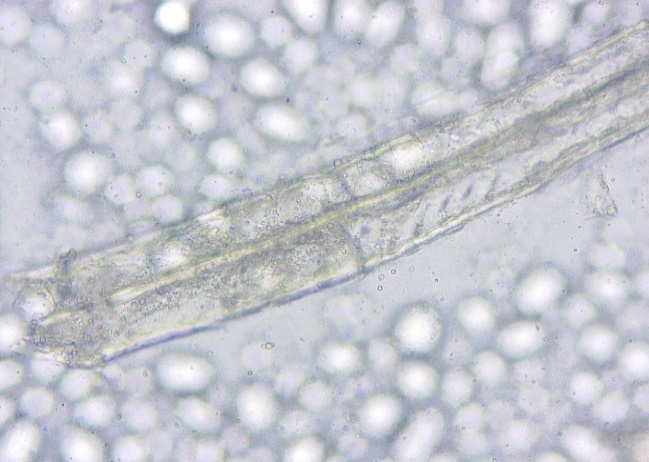 |
| --- | --- |
| Fragment of cork cells in surface view | Simple fibre |
| 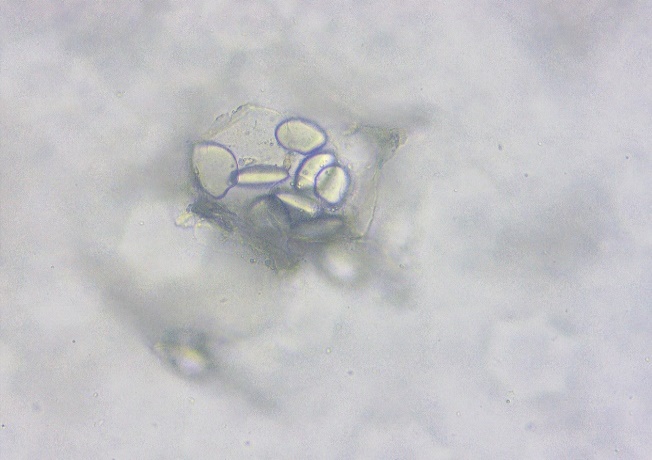 | 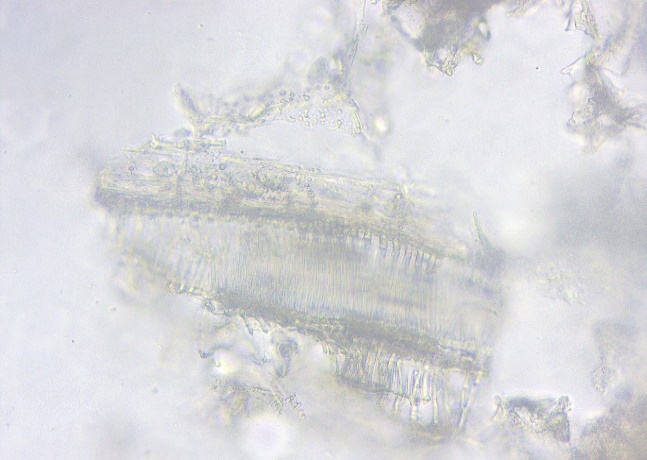 |
| Parenchyma cells with starch grains | Scalariform Vessels |
| Photomicrographs of powder microscopy of *Shunthi* Rhizome | |

**Table – .** **Physico-chemical, safety parameters and HPTLC analysis of *Shunthi* Rhizome.**

| **S. No.** | **Test(s)** | **Results (n=3)** |
| --- | --- | --- |
|  | Physical Test | Appearance: Ovate & flattish  Color: creamish yellow  Odor: Aromatic  Taste: Agreeable Pungent |
|  | Foreign Matter (% w/w) | 0.20 |
|  | Loss on Drying (% w/w) | 3.25 |
|  | pH (10% solution) | 4.10 |
|  | Water Extractive Value (% w/w) | 14.69 |
|  | Alcohol Extractive Value (% w/w) | 10.78 |
|  | Total Ash Value (% w/w) | 5.06 |
|  | Acid Insoluble Ash Value (% w/w) | 0.520 |
|  | Test for Heavy Metals (ppm)  Pb, Cd, Hg, As | Complies as per API |
|  | Aflatoxins | Complies as per API |
|  | Specific Pathogens | Complies as per API |
|  | Pesticides Residues | Complies as per API |
|  | TLC (R_f_ values)  “**HPTLC Analysis Report**”  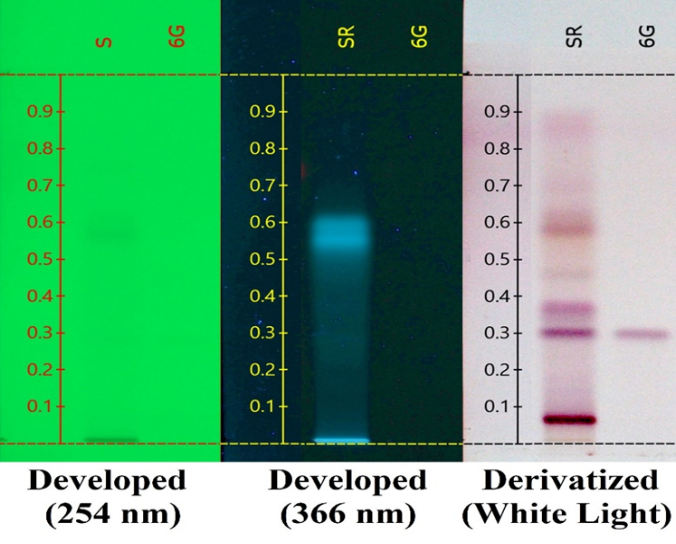 | Developing Solvent: Toluene: Ethyl Acetate (3: 1)  **SR**  At 254 nm: 0.008, 0.280, 0.570  At 366 nm: 0.008, 0.554, 0.735  Derivatized: 0.007, 0.055, 0.299, 0.372, 0.458, 0.572, 0.734  **Marker:** - 0.295 (6-gingerol)  The chromatogram obtained with test solution shows a band at Rf ≈ 0.299 corresponding to that of 6-gingerol (Rf ≈ 0.295).  Content (%) of **6-gingerol** = 0.28 % |

**Nagvalli (*Piper betel* Linn) Leaf**

1. **A. Organoleptic characters:** Powder of Nagvalli (*Piper betel* Linn) is greenish in colour with aromatic odour; pungent and astringent taste.

| Characteristic/Name of the Raw Drug | Nagvalli (*Piper betel* Linn) |
| --- | --- |
| Colour | Green |
| Odour | Aromatic |
| Taste | Pungent, Astringent |
| Texture | Smooth |

1. **Powder microscopy**

Powder microscopy of sample shows presence of epidermal cells with anisocytic stomata, fragments of epidermal cells in surface view, multicellular covering trichomes and spiral vessels. All the observed characters comply with the Ayurvedic Pharmacopoeia of India, Part I, Vol III, Monograph no. 28.

| 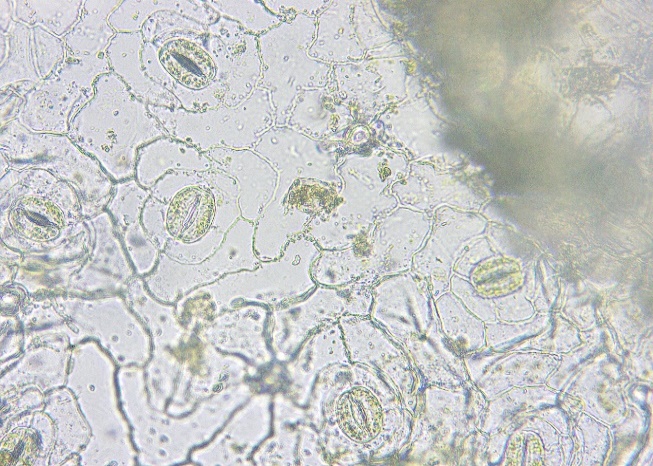 | 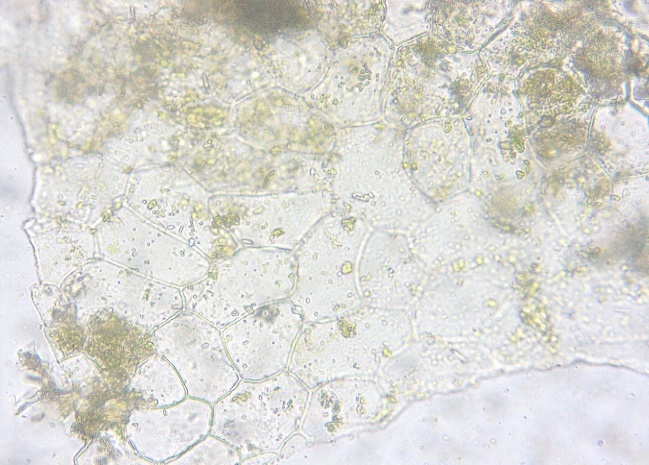 |
| --- | --- |
| Anisocytic stomata | Epidermal cells |
| 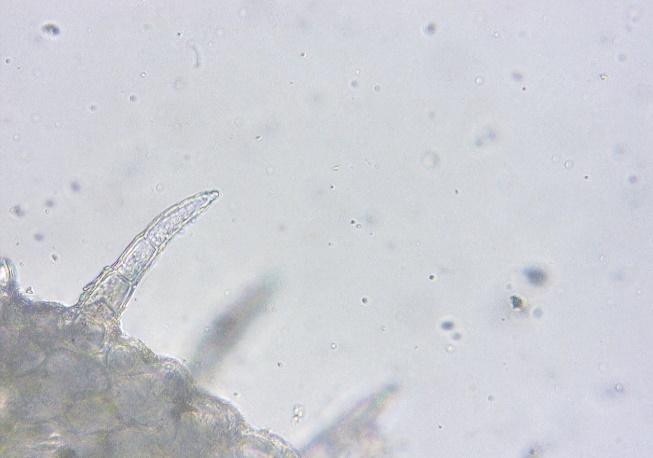 | 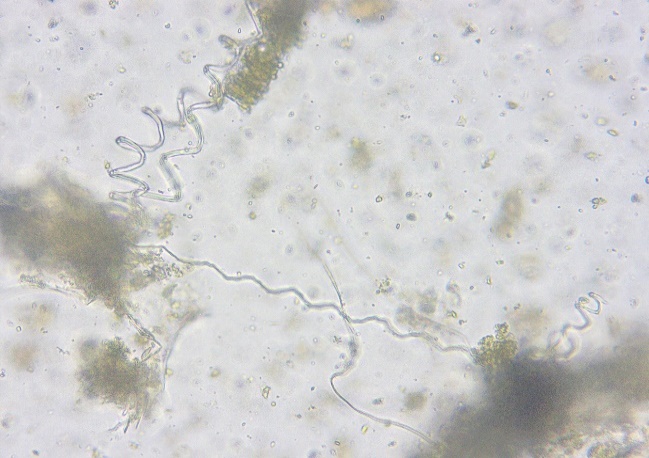 |
| Multicellular trichome | Spiral vessels |
| Photomicrographs of powder microscopy of Nagvalli (*Piper betel* Linn) leaf | |

**Table – . Physico-chemical and HPTLC analysis of *Nagvalli (Piper betel* Linn) fresh leaf.**

| **S. No.** | **Test(s)** | **Results (n=3)** |
| --- | --- | --- |
|  | Foreign Matter (% w/w) | ND |
|  | Loss on Drying (% w/w) | 2.06 |
|  | pH (10% solution) | 4.70 |
|  | Water Extractive Value (% w/w) | 20.6 |
|  | Alcohol Extractive Value (% w/w) | 18.5 |
|  | Total Ash Value (% w/w) | 16.73 |
|  | Acid Insoluble Ash Value (% w/w) | 1.07 |
|  | Test for Heavy Metals (ppm)  Pb, Cd, Hg, As | Complies as per API |
|  | Aflatoxins | Complies as per API |
|  | Specific Pathogens | Complies as per API |
|  | Pesticides Residues | Complies as per API |
|  | HPTLC (R_f_ values)  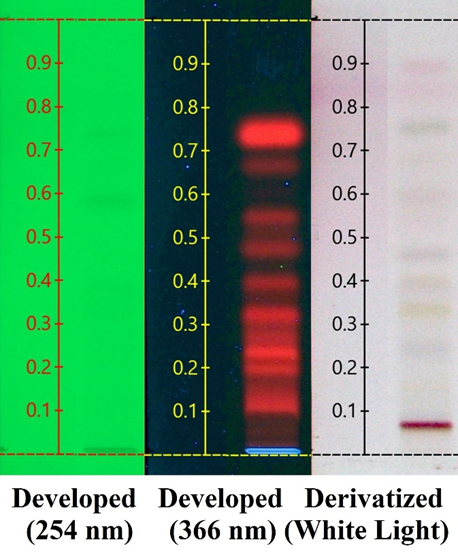 | Developing Solvent: Toluene: Ethyl Acetate (3: 1)  At 254 nm: 0.011, 0.381, 0.581, 0.737  At 366 nm: 0.011, 0.112, 0.197, 0.235, 0.320, 0.395, 0.474, 0.546, 0.666, 0.738  Derivatized: 0.010, 0.065, 0.240, 0.336, 0.387, 0.460, 0.592, 0.675, 0.754, 0.891 |

**Table.** Physicochemical Analysis of Goghrita (Clarified Butter).

| **S. No.** | **Test(s)** | **Results (n=3)** |
| --- | --- | --- |
|  | Brand | Anik Ghee |
|  | Rancidity | Negative |
|  | Moisture content | 0.0659 |
|  | Specific gravity (30^0^C) | 0.9338 |
|  | Refractive Index (40^0^C) | 1.457 |
|  | Iodine Value | 34.26 |
|  | Saponification Value | 198.44 |
|  | Acid Value | 0.59 |
|  | Carotene (IU) | 9840 |
|  | Peroxide Value | 2.62 |
|  | Unsaponifiable matter (%w/w) | 0.57 |
|  | Congealing Point (^0^C) | 18.5 |
|  | Viscosity (mPas) ((39^0^C)) | 9.31 |
|  | pH (10 % w/v) (32^0^C) | 4.302 |
|  | Test for Heavy Metals (ppm)  Pb, Cd, Hg, As | Complies as per API |
|  | Aflatoxins | Complies as per API |
|  | Specific Pathogens | Complies as per API |
|  | Pesticides Residues | Complies as per API |

**Raw Gairika (Red Ochre)**

1. **Physical Properties Raw Gairika (Red Ochre) (RG):**

| **S. No.** | **Test(s)** | **Results** |
| --- | --- | --- |
|  | Nature | Massive clayey |
|  | Colour | Reddish brown |
|  | Streak | Reddish brown |
|  | Cleavage | Not present |
|  | Fracture | Uneven |
|  | Lustre | Earthy |
|  | Tenacity | Brittle |
|  | Transparency | Opaque |
|  | Hardness | 6.2 |
|  | Sp. Gr | 2.7 |
|  | Magnetism | Magnetic effect |
|  | Effect of heat | No change occur, shows magnetic effect on heating |
|  | Solubility in water | Insoluble but soluble in HCl. |

**Table – 2. Physico-chemical, safety parameters of Raw Gairika (Red Ochre) (RG)**

| **S. No.** | **Test(s)** | **Results (n=3)** |
| --- | --- | --- |
|  | Loss on ignition (% w/w) | 11.35 |
|  | Silica (SiO_2_) | 1.40 |
|  | Test for Heavy Metals (ppm) Pb, Cd, Hg, As | Complies as per API |
|  | Aflatoxins | Complies as per API |
|  | Specific Pathogens | Complies as per API |
|  | Pesticides Residues | Complies as per API |
|  | Test for elements | |
|  | Na (ppm) | 1377.25 |
|  | K (ppm) | 139.72 |
|  | Ca (ppm) | 256.64 |
|  | Mg (ppm) | 34.96 |
|  | Al (ppm) | 18138.4 |
|  | Fe (ppm) | 198617.37 |

**Table .** Physicochemical Analysis of shodita (purified) Gairika (SRG).

| **S. No.** | **Test(s)** | **Results (n=3)** | | |
| --- | --- | --- | --- | --- |
|  |  | **SRG -I** | **SRG -II** | **SRG -III** |
|  | Loss on ignition (% w/w) | 14.38 | 14.33 | 14.33 |
|  | Silica (SiO_2_) | 1.81 | 1.81 | 1.81 |
|  | Test for Heavy Metals (Pb, Cd, Hg, As in ppm) | Complies as per API | | |
|  | Aflatoxins | Complies as per API | | |
|  | Specific Pathogens | Complies as per API | | |
|  | Pesticides Residues | Complies as per API | | |
|  | Test for elements | | | |
|  | Na (ppm) | 229.40 | 229.40 | 228.4 |
|  | K (ppm) | 100.01 | 99.80 | 99.54 |
|  | Ca (ppm) | 374.67 | 374.67 | 375.43 |
|  | Mg (ppm) | 32.14 | 32.18 | 32.19 |
|  | Al (ppm) | 10834.78 | 10834.74 | 10798.65 |
|  | Fe (ppm) | 141023.63 | 141023.63 | 141023.63 |

**Table .** Physicochemical Analysis (in-process IPQC) of LSR granules (GLSR).

| **S. No.** | **Test(s)** | **Results (n=3)** | | |
| --- | --- | --- | --- | --- |
|  |  | **GLSR-1** | **GLSR-II** | **GLSR-II** |
|  | Loss on Drying (% w/w) | 4.56 | 4.72 | 4.65 |
|  | pH (10% aqueous solution) | 5.213 | 5.376 | 5.182 |
|  | Water Extractive Value (% w/w) | 29.54 | 31.55 | 30.24 |
|  | Alcohol Extractive Value (% w/w) | 10.11 | 9.64 | 10.90 |
|  | Total Ash Value (% w/w) | 27.17 | 26.69 | 28.11 |
|  | Acid Insoluble Ash Value (% w/w) | 20.88 | 22.11 | 22.27 |
|  | Bulk Density (g/cm^3^) | 0.7317 | 0.7407 | 0.7652 |
|  | Tap Density (g/cm^3^) | 0.9013 | 0.8924 | 0.9002 |
|  | Hausner Ratio | 1.1958 | 1.2048 | 1.1765 |
|  | Compressibility Index (%) | 16.000 | 17.000 | 15.650 |
|  | Na (ppm) | 1814.22 | 1739.72 | 1757.32 |
|  | K (ppm) | 6884.05 | 6722.48 | 6879.40 |
|  | Ca (ppm) | 4075.35 | 4135.12 | 4135.04 |
|  | Mg (ppm) | 2617.93 | 2619.57 | 2579.84 |
|  | Al (ppm) | 20882.27 | 21120.45 | 20265.37 |
|  | Fe (ppm) | 33098.16 | 33408.07 | 31919.66 |
|  | Test for Heavy Metals (ppm)  (Pb, Cd, Hg, As) | Complies as per API | | |
|  | Aflatoxins | Complies as per API | | |
|  | Specific Pathogens | Complies as per API | | |
|  | Pesticides Residues | Complies as per API | | |

**Laghu Sutashekhar Rasa (LSR): Goghrita Shodhita Gairika (Purified Red ochre processed in clarified butter)**

**A. Organoleptic characters:** A reddish or off reddish color tablet of spherical or biconvex shape, with Ghrita-like odour, metallic taste and smooth in texture.

| **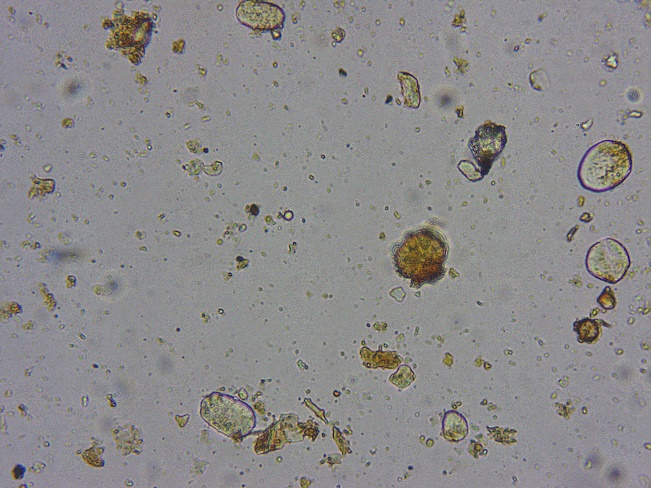** | **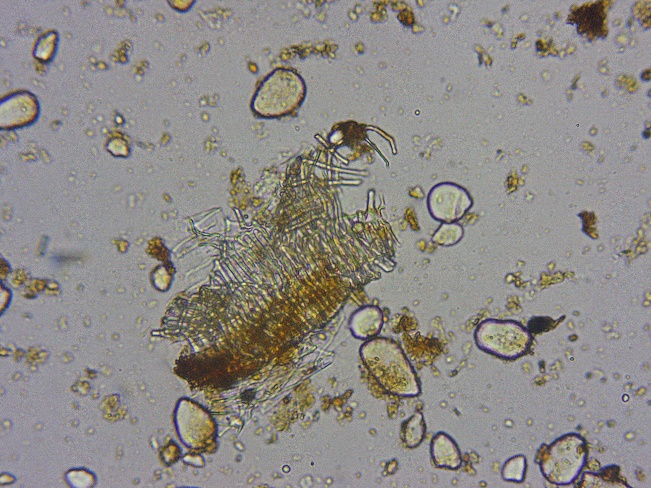** |
| --- | --- |
| Oil globules *(Ghrita)* | Scalariform vessels *(Shunthi)* |
| **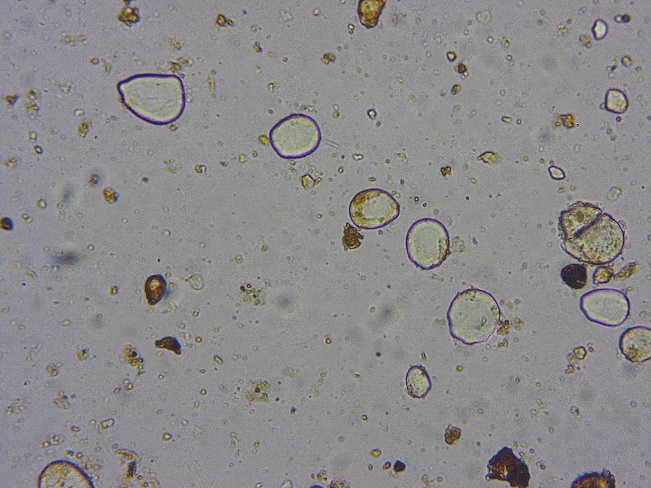** | **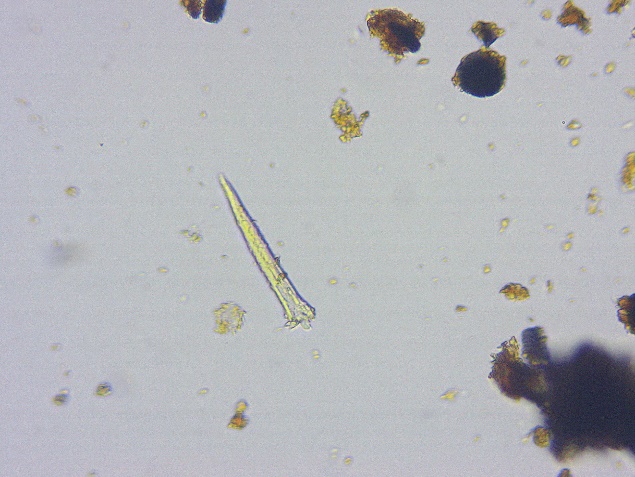** |
| Starch grains *(Shunthi)* | Trichome (*Nagvalli*) |
| Photomicrographs of powder microscopy of Laghu Sutashekhar Rasa (LSR) | |

**Table - 1.** Physicochemical Analysis of LSR tablets.

| **S. No.** | **Test(s)** | **LSR-I** | **LSR-II** | **LSR-III** |
| --- | --- | --- | --- | --- |
|  | Size | Dia. 9.61 ± 0.14 mm Thickness 4.76 ± 0.08 mm | | |
|  | Foreign Matter (% w/w) | **Nil** | **Nil** | **Nil** |
|  | Loss on Drying (% w/w) | 3.87 | 3.55 | 3.69 |
|  | pH (10% aqueous solution) | 5.327 | 5.361 | 5.331 |
|  | Water Extractive Value (% w/w) | 44.52 | 43.08 | 45.07 |
|  | Alcohol Extractive Value (% w/w) | 12.54 | 10.87 | 11.88 |
|  | Total Ash Value (% w/w) | 21.98 | 22.28 | 22.42 |
|  | Acid Insoluble Ash Value (% w/w) | 17.70 | 17.62 | 18.13 |
|  | Average Weight (g) | 0.40267 | 0.40343 | 0.40549 |
|  | Hardness (kg/cm^2^) | 4.34 | 4.40 | 4.75 |
|  | Disintegration Time (min.) | 17:16 | 17:21 | 17:43 |
|  | Friability (% w/w) | 0.45 | 0.46 | 0.46 |
|  | Na (ppm) | 2214.87 | 1992.85 | 2124.55 |
|  | K (ppm) | 7925.10 | 8078.63 | 7844.19 |
|  | Ca (ppm) | 7912.22 | 8595.75 | 8213.48 |
|  | Mg (ppm) | 3178.78 | 2789.08 | 2621.91 |
|  | Al (ppm) | 23217.84 | 23006.11 | 23603.59 |
|  | Fe (ppm) | 31583.18 | 31432.04 | 30645.45 |
|  | Test for Heavy Metals (ppm)  (Pb, Cd, Hg, As) | Complies as per API | | |
|  | Aflatoxins | Complies as per API | | |
|  | Specific Pathogens | Complies as per API | | |
|  | Pesticides Residues | Complies as per API | | |
